# New perspectives on the annihilation electrogenerated chemiluminescence of mixed metal complexes in solution†
†Electronic supplementary information (ESI) available: Additional electrochemical, spectroscopic, synthetic, and ECL data, as described in the main article text. See DOI: 10.1039/c6sc01570k


**DOI:** 10.1039/c6sc01570k

**Published:** 2016-04-29

**Authors:** Emily Kerr, Egan H. Doeven, Gregory J. Barbante, Conor F. Hogan, David J. Hayne, Paul S. Donnelly, Paul S. Francis

**Affiliations:** a Centre for Chemistry and Biotechnology , School of Life and Environmental Sciences , Faculty of Science , Engineering and Built Environment , Deakin University , Geelong , Victoria 3220 , Australia . Email: paul.francis@deakin.edu.au; b Centre for Regional and Rural Futures , School of Life and Environmental Sciences , Faculty of Science , Engineering and Built Environment , Deakin University , Geelong , Victoria 3220 , Australia . Email: egan.doeven@deakin.edu.au; c Department of Chemistry and Physics , La Trobe Institute for Molecular Science , La Trobe University , Melbourne , Victoria 3086 , Australia; d School of Chemistry and Bio21 Molecular Science and Biotechnology Institute , University of Melbourne , Melbourne 3010 , Australia

## Abstract

Preliminary explorations of the annihilation electrogenerated chemiluminescence (ECL) of mixed metal complexes have revealed opportunities to enhance emission intensities and control the relative intensities from multiple luminophores through the applied potentials. However, the mechanisms of these systems are only poorly understood. Herein, we present a comprehensive characterisation of the annihilation ECL of mixtures of tris(2,2′-bipyridine)ruthenium(ii) hexafluorophosphate ([Ru(bpy)_3_](PF_6_)_2_) and *fac*-tris(2-phenylpyridine)iridium(iii) ([Ir(ppy)_3_]). This includes a detailed investigation of the change in emission intensity from each luminophore as a function of both the applied electrochemical potentials and the relative concentrations of the two complexes, and a direct comparison with two mixed (Ru/Ir) ECL systems for which emission from only the ruthenium-complex was previously reported. Concomitant emission from both luminophores was observed in all three systems, but only when: (1) the applied potentials were sufficient to generate the intermediates required to form the electronically excited state of both complexes; and (2) the concentration of the iridium complex (relative to the ruthenium complex) was sufficient to overcome quenching processes. Both enhancement and quenching of the ECL of the ruthenium complex was observed, depending on the experimental conditions. The observations were rationalised through several complementary mechanisms, including resonance energy transfer and various energetically favourable electron-transfer pathways.

## Abstract 2

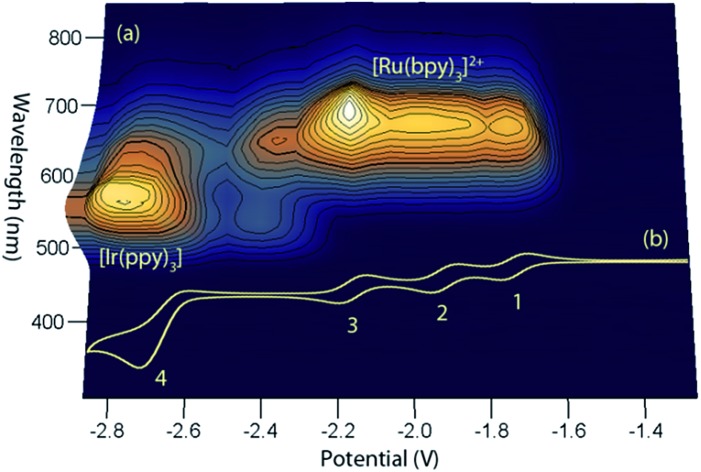
We examine energy transfer and quenching within annihilation ECL systems comprising mixed metal-complexes in solution, and show the dependence of the emission intensities on luminophore concentration and the applied potentials.

## Introduction

Electrogenerated chemiluminescence (also known as electrochemiluminescence or ECL) is the emission of light resulting from reactions between electrochemically generated species.^1^,^2^ ECL is a consequence of the so-called ‘inverted region’ of Marcus electron transfer theory.^3^,^4^ It transpires because the energy available from the homogeneous electron-transfer processes is too large to be dissipated on the timescale of the vibrational modes of the emitter's molecular framework.

ECL is often categorised into two general pathways: annihilation and co-reactant.^1^ Annihilation ECL involves the direct electrochemical formation of both oxidised and reduced species, normally as a result two-directional potential stepping. These oxidised and reduced species may then react to form electronically excited products capable of emitting light, as shown in reactions (1)–(4), where A and D may be the same or in the case of ‘mixed’ ECL systems, different molecules.

1 A + e^–^ → A˙^–^

2 D – e^–^ → D˙^+^

3a A˙^–^ + D˙^+^ → A + D*

3b A˙^–^ + D˙^+^ → A* + D

3c A˙^–^ + D˙^+^ → A + D

4a D* → D + *hν*

4b A* → A + *hν*

For annihilation ECL systems, in most cases the Gibbs free energy associated with the formation of either ground (Δ*G*_gs_) (reaction (3c)) or excited (Δ*G*_es_) state (reactions (3a) or (3b)) products can be reasonably estimated from the respective electrochemical potentials and the emission energy, as shown in eqn (I) and (II) (with further details in the ESI†).^4^–^6^

I Δ*G*_gs_ ≈ *E*°_D+/D_ – *E*°_A/A–_

IIa Δ*G*_es_(D*) ≈ (*E*°_D+/D_ – *E*°_A/A–_) + *E*_es_(D*)

IIb Δ*G*_es_(A*) ≈ (*E*°_D+/D_ – *E*°_A/A–_) + *E*_es_(A*)

where *E*_es_(D*) and *E*_es_(A*) are the excited-state energies of complexes A and D, obtained from their photoluminescence emission spectra. For simplicity, minor contributions from factors such as the Coulomb repulsion of bringing the reactants together and the Franck–Condon energy of the emissive product have been omitted from these equations.^6^,^7^

The question of whether the oxidised or reduced partner becomes the electronically excited species responsible for the emission (*i.e.* whether the system proceeds through reactions (3a) and (4a) or reactions (3b) and (4b)) is important from both a fundamental and a practical standpoint.^6^,^8^,^9^ In the classic tris(2,2′-bipyridine)ruthenium(ii) ([Ru(bpy)_3_]^2+^) annihilation ECL system,^10^ for example, reactants A and D are both [Ru(bpy)_3_]^2+^, and therefore reactions (3a) and (3b) are thermodynamically equivalent. Simple orbital overlap arguments, however, suggest that ligand-to-ligand electron transfer to form [Ru(bpy)_3_]^2+^* from the oxidised parent will be kinetically favoured over metal-to-metal electron transfer to form the excited state from the reduced parent.^11^ Nevertheless, co-reactant ‘oxidative–reduction’ and ‘reductive–oxidation’ ECL pathways^12^,^13^ show that either the oxidised or reduced intermediate can become the emitting species.

Mixed annihilation ECL systems can be more complicated, as the oxidation and/or reduction of both reactants, and two possible emitting species, need to be considered.^14^ Nevertheless, the emission spectra of many mixed ECL systems comprising organic reactants have shown that these reactions almost always generate a single emitting species,^5^ which can be attributed to the rates of the competing electron transfer processes and the relative energies of the possible excited states. Mixed systems that combine a transition metal complex with an organic compound have been used to generate exceptionally high annihilation ECL efficiencies from the metal complex.^15^

In our recent preliminary report of the mixed annihilation ECL of transition metal complexes (combining [Ru(bpy)_3_]^2+^ with a variety of iridium(iii) complexes),^6^ we observed simultaneous emissions from multiple emitters, and showed that the ratio of these emissions (and hence the overall colour of the luminescence) could be tuned though the applied electrode potentials, exploiting the multiple, closely spaced reductions and oxidations of the reactants. In a subsequent study, Swanick *et al.*^9^ examined the ECL of a ruthenium(ii)–iridium(iii) complex ‘soft salt’,^16^ comprising a [Ru(dtb-bpy)_3_]^2+^ cation (where dtb-bpy = 4,4′-di-*t*-butyl-2,2′-bipyridine) and two [Ir(ppy)_2_(CN)_2_]^–^ anions (where ppy = 2-phenylpyridine), in solution. In an unanticipated result,^9^ unlike the case with the photoluminescence of the soft salt under closely related conditions,^17^ Swanick *et al.* observed ECL solely from the ruthenium(ii) complex (*i.e.*, no emission from the iridium(iii) complex). This contrasts with our reports of multiple ECL emissions from mixtures of ruthenium(ii) and iridium(iii) complexes involving annihilation^6^ or co-reactant^13^,^18^,^19^ ECL pathways. Swanick *et al.*^9^ attributed the absence of ECL from the iridium(iii) luminophore (and enhancement of the [Ru(dtb-bpy)_3_]^2+^ emission) to the rapid consumption of electrochemically reduced iridium species through electron transfer to the ruthenium complex, which precluded the formation of the iridium(iii) emitter. Similarly, seeking to fabricate a colour-tuneable ECL-based light-emitting device,^8^ Moon *et al.* incorporated [Ru(bpy)_3_]^2+^ and [Ir(df-ppy)_2_(bpy)]^+^ (where df-ppy = 2-(2,4-difluorophenyl)pyridine) into an ion-gel cast onto an ITO-coated flexible substrate. ECL was observed from only the ruthenium(ii) complex, but the inclusion of the iridium(iii) species was found to enhance the emission intensity up to 2-fold. In this case, the absence of ECL from the iridium(iii) complex was attributed to electron transfer quenching of the excited state.

Herein, we reconcile these seemingly disparate findings through an examination of concentration effects and energy transfer in mixed annihilation ECL, whilst introducing both a novel three-dimensional representation of the phenomenon (annihilation ECL intensity *versus* emission wavelength and the applied reduction potential) and a simple graphical depiction of the energetics of annihilation and co-reactant ECL systems to explore electron-transfer quenching pathways.

## Experimental

### Chemicals

Acetonitrile (Ajax Finechem, Australia) was distilled over calcium hydride under nitrogen and solutions were degassed with grade 5 argon prior to analysis. Tetrabutylammonium hexafluorophosphate ([TBA][PF_6_], 99.5%, electrochemical grade) and *fac*-tris[2-(2-pyridinyl-κ*N*)phenyl-κ*C*]iridium (tris(2-phenylpyridinato-*C*^2^,*N*)iridium(iii); [Ir(ppy)_3_], 99%) were purchased from Sigma-Aldrich (Australia). Tris(2,2′-bipyridine)ruthenium(ii) dichloride hexahydrate ([Ru(bpy)_3_]Cl_2_·6H_2_O) was purchased from Strem Chemicals (USA) and converted to the hexafluorophosphate salt ([Ru(bpy)_3_](PF_6_)_2_). Tris[2-(1H-pyrazol-1-yl-κ*N*^2^)phenyl-κ*C*]iridium (tris(phenylpyrazole)iridium(iii); [Ir(ppz)_3_], >99%) was purchased from LumTech (Taiwan). Details of the synthesis and characterisation of [Ir(df-ppy)_2_(bpy)](PF_6_), [Ru(dtb-bpy)_3_](PF_6_)_2_, TBA[Ir(ppy)_2_(CN)_2_], and the [Ru(dtb-bpy)_3_][Ir(ppy)_2_(CN)_2_]_2_ soft salt are included in the ESI.†

### Experimental procedure

An Autolab PGSTAT 101 or PGSTAT 128N potentiostat was used to perform chronoamperometry and cyclic voltammetry experiments (Metrohm Autolab B.V., Netherlands). The instrumental configuration was equivalent to that described previously.^20^ For cyclic voltammetry measurements, the complexes were prepared at 0.25 mM in degassed, freshly distilled acetonitrile (0.1 M [TBA][PF_6_] supporting electrolyte) and referenced to the formal potential of the ferrocene/ferrocenium couple (1 mM), measured *in situ* in each case. ECL spectra were obtained using a model QE65pro CCD spectrometer (Ocean Optics) interfaced with the working electrode through a collimating lens and custom built cell holder (Fig. S1 in ESI†); the potentiostat applied a two-step chronoamperometry pulse at 0.5 Hz (*i.e.* alternating 1 s oxidative potential with 1 s reductive potential) for 12 s, unless otherwise stated. Intensities were calculated from the average integrated peak area of three replicates. For convenience, the arbitrary intensity units from spectrometer were divided by 10^3^. To generate the 3D profiles (intensity *versus* emission wavelength and applied reduction potential) of annihilation ECL, appropriate concentrations of the complexes were prepared in freshly distilled acetonitrile with 0.1 M [TBA][PF_6_] supporting electrolyte, and solutions were degassed with grade 5 argon prior to analysis. NOVA software was configured to apply a two-step 0.5 Hz pulse from the oxidative potential to corresponding reduction potentials, for 12 s, with a 30 s wait time between each pulse sequence, to allow for degassing (15 s) between the collection of each spectrum.

## Results and discussion

### The [Ru(bpy)_3_]^2+^–[Ir(ppy)_3_] mixed annihilation ECL system

**Fig. 1:**
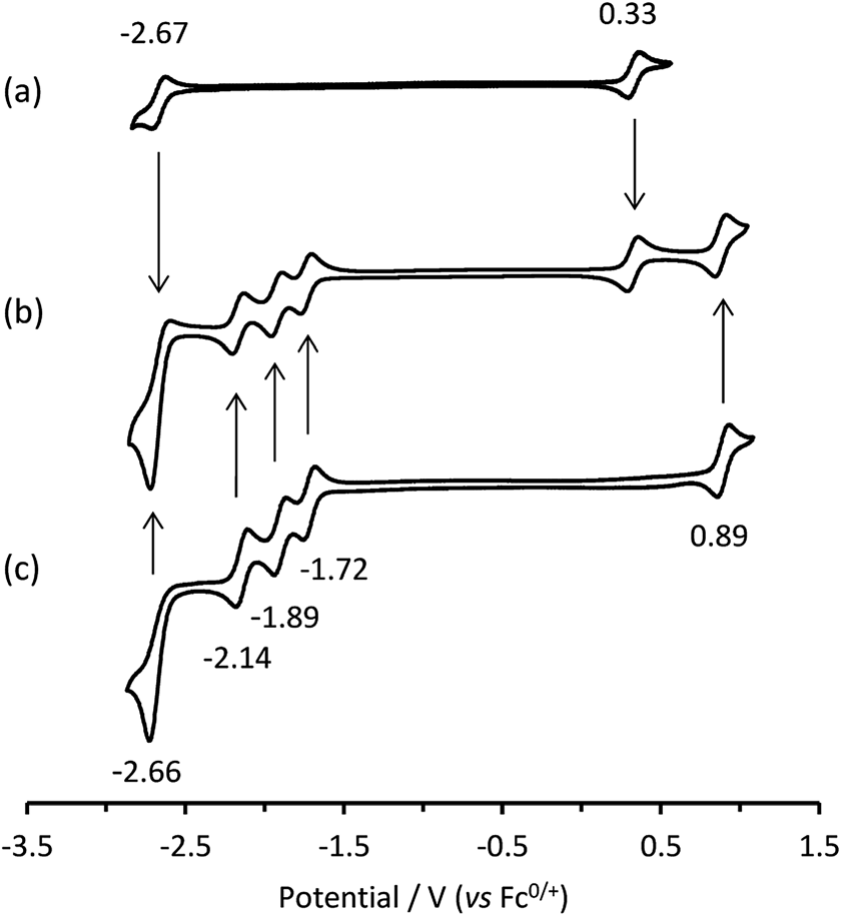
Cyclic voltammograms of: (a) [Ir(ppy)_3_]; (b) a mixture of [Ir(ppy)_3_] and [Ru(bpy)_3_]^2+^; and (c) [Ru(bpy)_3_]^2+^, showing *E*° values. All complexes at 0.25 mM with 0.1 M [TBA][PF_6_] supporting electrolyte in acetonitrile. Scan rate: 0.1 V s^–1^.

Cyclic voltammetric scans of an equimolar mixture of [Ru(bpy)_3_]^2+^ and [Ir(ppy)_3_] in acetonitrile containing 0.1 M [TBA][PF_6_] (Fig. 1b) exhibit a combination of the characteristic electron-transfer processes of the two metal complexes (Fig. 1a and c).

In our previous report of annihilation ECL from mixtures of [Ru(bpy)_3_]^2+^ with various iridium(iii) complexes (including [Ir(ppy)_3_]),^6^ we observed, under certain circumstances, simultaneous emissions from both luminophores. Moreover, the relative intensity of the emissions could be manipulated through the applied voltages, which generated different redox forms of the complexes, thus modifying the energetics of the light-producing reactions. For example, when alternately pulsing slightly beyond the first reduction potential of [Ru(bpy)_3_]^2+^ and the oxidation potential of [Ir(ppy)_3_], we form [Ru(bpy)_3_]^+^ and [Ir(ppy)_3_]^+^ (but at these potentials, neither [Ir(ppy)_3_]^–^ nor [Ru(bpy)_3_]^3+^ is formed). Estimations of the Δ*G* of the subsequent reaction between [Ru(bpy)_3_]^+^ and [Ir(ppy)_3_]^+^ (eqn (IIa) and (IIb)) indicated that the generation of [Ru(bpy)_3_]^2+^* and [Ir(ppy)_3_] (reaction (5)) was energetically favourable, but there was insufficient energy to produce [Ir(ppy)_3_]* and [Ru(bpy)_3_]^2+^. Under these conditions, the characteristic orange-red emission of the [Ru(bpy)_3_]^2+^ complex was observed. However, by pulsing to further negative potentials, more reductive intermediates were formed, which upon reaction with [Ir(ppy)_3_]^+^, enabled the [Ir(ppy)_3_]* species to be attained (Δ*G* < 0). Pulsing beyond the third reduction potential of [Ru(bpy)_3_]^2+^ and the oxidation potential of [Ir(ppy)_3_] gave an overall yellow emission from a combination of emissions from reactions (5) and (6). Whereas pulsing beyond the reduction and oxidation potentials of [Ir(ppy)_3_] gave the characteristic green emission of the [Ir(ppy)_3_] complex predominantly *via* reaction (7).

5 [Ru(bpy)_3_]^+^ + [Ir(ppy)_3_]^+^ → [Ru(bpy)_3_]^2+^* + [Ir(ppy)_3_]

6 [Ru(bpy)_3_]^–^ + [Ir(ppy)_3_]^+^ → [Ru(bpy)_3_] + [Ir(ppy)_3_]*

7 [Ir(ppy)_3_]^–^ + [Ir(ppy)_3_]^+^ → [Ir(ppy)_3_]* + [Ir(ppy)_3_]

**Fig. 2:**
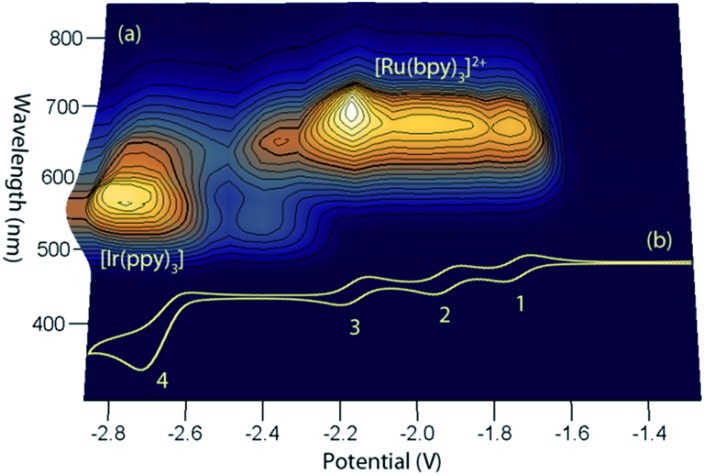
(a) A 3D representation of the ECL of the [Ru(bpy)_3_]^2+^–[Ir(ppy)_3_] mixed annihilation system showing ECL intensity *versus* emission wavelength and applied reductive potential, with an alternating oxidative potential of 0.98 V (to generate both [Ir(ppy)_3_]^+^ and [Ru(bpy)_3_]^3+^), using 0.01 mM [Ru(bpy)_3_]^2+^ and 0.24 mM [Ir(ppy)_3_] in acetonitrile with 0.1 M [TBA][PF_6_]. A similar graph was obtained using an oxidative potential of 0.43 V, which generated [Ir(ppy)_3_]^+^, but not [Ru(bpy)_3_]^3+^ (Fig. S2†). (b) The corresponding portion of a cyclic voltammogram of 0.25 mM [Ru(bpy)_3_]^2+^ and 0.25 mM [Ir(ppy)_3_] (0.1 M [TBA][PF_6_], acetonitrile), showing: (1) [Ru(bpy)_3_]^1+/2+^; (2) [Ru(bpy)_3_]^0/1+^; (3) [Ru(bpy)_3_]^1–/0^; (4) a combination of [Ir(ppy)_3_]^1–/0^ and [Ru(bpy)_3_]^2–/1–^. All potentials shown *versus* the Fc^0/+^ redox couple.

This simple comparison of the ECL generated at a few sets of applied potentials shows that free energy considerations (eqn (II)) provide a basis for understanding the potential dependence of the ECL in observed in such mixed annihilation systems, but it is an incomplete characterisation due to the possibility of other ground state and excited state interactions between the species that are present. With this in mind, we adapted our 3D ECL approach that was previously used to examine the parameters of mixed co-reactant ECL systems.^13^,^19^ This involved an automated pulsing cycle from 100 mV beyond a single oxidative potential to a series of evenly spaced reductive potentials at 50 mV intervals over the range of interest, whilst monitoring the ECL spectra with a CCD spectrometer (Fig. 2).

The relevant portion of the cyclic voltammogram was superimposed on the graph so that the electrochemical generation of various reduced species could be easily correlated with the emission processes. In agreement with our previous results,^6^ homogeneous electron transfer to [Ir(ppy)_3_]^+^ generates the [Ir(ppy)_3_]* emitter, but only with reducing agents at least as strong as [Ru(bpy)_3_]^–^, and the relative emission intensities from [Ru(bpy)_3_]^2+^* and [Ir(ppy)_3_]* thereafter were highly dependent on the applied potential.

In our previous study,^6^ we sought concentrations of the two complexes that would generate similar ECL intensities, to demonstrate the control of the emission ratio (and overall emission colour) through the applied potentials. In ECL system shown in Fig. 2a, the ratio of iridium to ruthenium complex is 24 : 1, which is much greater than those used in the studies by Moon *et al.*^8^ (up to 4 : 1) and Swanick *et al.*^9^ (2 : 1), in which ECL from only one luminophore was observed.

**Fig. 3:**
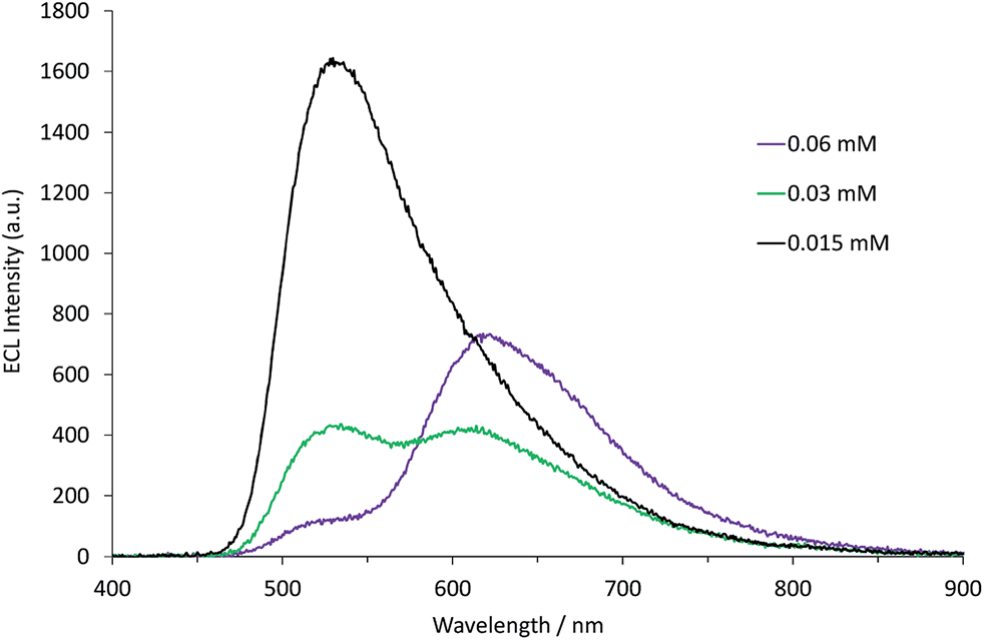
Annihilation ECL spectra (0.99 V to –2.77 V *vs.* Fc^0/+^) for a mixture of [Ir(ppy)_3_] (0.25 mM) and [Ru(bpy)_3_]^2+^ (0.015–0.060 mM) in acetonitrile with 0.1 M [TBA][PF_6_]. For each spectrum, a two-step potential pulse was applied at 0.5 Hz for 12 s.

Applying the above comprehensive approach to explore the [Ru(bpy)_3_]^2+^–[Ir(ppy)_3_] mixed annihilation ECL system at a range of metal complex concentrations (*e.g.*, Fig. S3†) confirmed that the contrasting observations of these previous studies^6^,^8^,^9^ can be largely ascribed to differences in the relative concentration of electrochemiluminophores. For example, under the conditions shown in Fig. 2, the ECL obtained when applying potentials 100 mV beyond the oxidation of [Ru(bpy)_3_]^2+^ (0.89 V *vs.* Fc^0/+^) and the reduction of [Ir(ppy)_3_] (–2.67 V *vs.* Fc^0/+^) arises predominantly (but not entirely) from the [Ir(ppy)_3_]* emitter. However, as the concentration of [Ru(bpy)_3_]^2+^ in the mixture was increased, we observed an increase in the characteristic emission from [Ru(bpy)_3_]^2+^* and decrease from [Ir(ppy)_3_]* (Fig. 3).

### Energy transfer in mixed annihilation ECL systems

Prior photoluminescence studies of the [Ru(dtb-bpy)_3_][Ir(ppy)_2_(CN)_2_]_2_ soft salt indicated Förster (resonance) energy transfer between the donor (Ir) and acceptor (Ru) complexes, with considerable overlap between their MLCT emission and absorption bands.^17^ However, electron transfer between ground and excited states of the complexes could not be ruled out in this system,^17^ and this process has been ascribed as the major pathway for energy transfer in photoluminescence studies of related soft salts (such as [Ir(Me-ppy)_2_(dtb-bpy)][Ir(df-ppy)_2_(CN)_2_]) that exhibit very little overlap between emission and absorption bands.^21^ In ECL experiments, significant quantities of the ground state oxidised and reduced species are generated, which must also be considered. The absence of iridium-based ECL from the [Ru(dtb-bpy)_3_][Ir(ppy)_2_(CN)_2_]_2_ soft salt, for example, was ascribed^9^ to electron transfer from the electrochemically reduced [Ir(ppy)_2_(CN)_2_]^2–^ species to [Ru(dtb-bpy)_3_]^2+^ (reaction (8)).

8 [Ir(ppy)_2_(CN)_2_]^2–^ + [Ru(dtb-bpy)_3_]^2+^ → [Ir(ppy)_2_(CN)_2_]^–^ + [Ru(dtb-bpy)_3_]^+^

In contrast, the absence of ECL from the iridium(iii) luminophore in mixtures of [Ru(bpy)_3_]Cl_2_ and [Ir(df-ppy)_2_(bpy)](PF_6_) was tentatively postulated^8^ to involve oxidative quenching of the electronically excited [Ir(df-ppy)_2_(bpy)]^+^* resulting in the direct formation of [Ru(bpy)_3_]^2+^* (reaction (9)).

9 [Ir(df-ppy)_2_(bpy)]^+^* + [Ru(bpy)_3_]^3+^ → [Ir(df-ppy)_2_(bpy)]^2+^ + [Ru(bpy)_3_]^2+^*

**Fig. 4:**
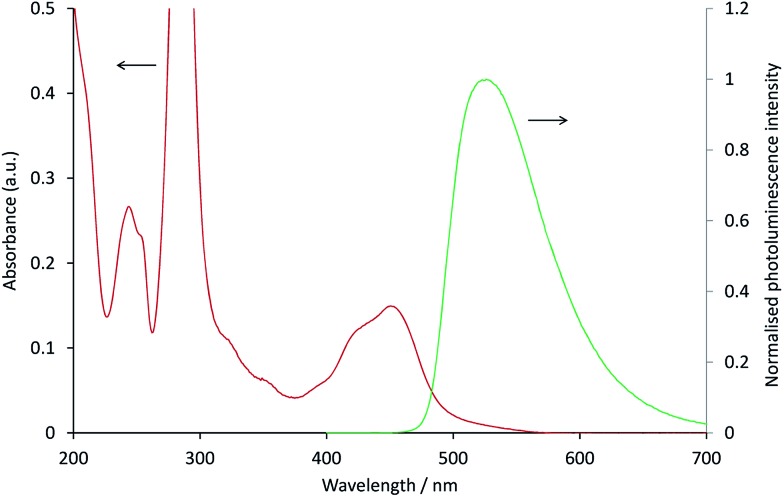
Absorption spectrum of 10 μM [Ru(bpy)_3_]^2+^ (red line) and photoluminescence emission spectrum (*λ*_ex_ = 380 nm) of 10 μM [Ir(ppy)_3_] (green line), in acetonitrile.

In the [Ru(bpy)_3_](PF_6_)_2_–[Ir(ppy)_3_] system, as with the system discussed above, the ECL of the iridium complex was efficiently quenched by the ruthenium-complex. The emission of [Ir(ppy)_3_] overlaps with the MLCT absorption band of [Ru(bpy)_3_]^2+^ (Fig. 4), but to a much lesser extent than that of [Ir(ppy)_2_(CN)_2_]^–^ and [Ru(dtb-bpy)_3_]^2+^,^17^ and therefore Förster resonance energy transfer could be anticipated to make only a minor contribution to the [Ru(bpy)_3_](PF_6_)_2_–[Ir(ppy)_3_] mixed annihilation ECL system.

**Fig. 5:**
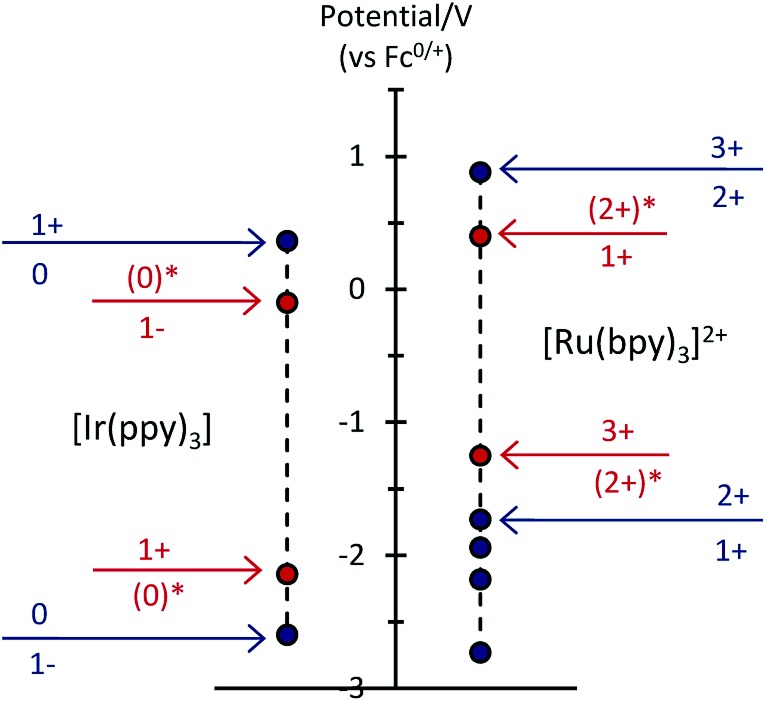
Redox potentials for ground states (blue dots) and electronically excited states (red dots) within the [Ru(bpy)_3_]^2+^–[Ir(ppy)_3_] mixed annihilation ECL system.

To examine the feasible electron transfer pathways, we plotted the electrochemical potentials (from Fig. 1) of both electrochemiluminophores and superimposed the corresponding potentials for their electronically excited states (Fig. 5 and S4a†), which have been estimated based on the low temperature (77 K) photoluminescence emission spectra.^22^ In this depiction, the species shown above the arrows at the top of the graph are the strongest oxidants and the species below the arrows at the bottom of the graph are the strongest reductants.

Considering first the electron transfer between the ground and excited states of the most stable oxidation state: the [Ir(ppy)_3_] complex in its ^3^MLCT excited state is a strong reductant that can donate an electron to [Ru(bpy)_3_]^2+^ (reaction (10) and Fig. S4b; † Δ*G* ≈ –0.41 eV). It can be estimated that the back electron transfer to generate the excited ruthenium complex (reaction (11)) is marginally energy insufficient (Δ*G* ≈ +0.04 eV), but it should be noted that (a) this is small compared to the combined estimation error of the excited state potentials and Δ*G*, and (b) the electron exchange may be a concerted process in which the overall energetics are favourable (Fig. S4c†).

10 [Ir(ppy)_3_]* + [Ru(bpy)_3_]^2+^ → [Ir(ppy)_3_]^+^ + [Ru(bpy)_3_]^+^

11 [Ir(ppy)_3_]^+^ + [Ru(bpy)_3_]^+^ → [Ir(ppy)_3_] + [Ru(bpy)_3_]^2+^*

The excitation process of annihilation ECL (unlike that of photoluminescence) generates significant quantities of the oxidised and reduced complexes (near the electrode surface) and therefore the contribution of these species to energy transfer must also be considered. Fig. 5 suggests a series of additional energetically feasible electron-transfers that may contribute to the observed quenching of the [Ir(ppy)_3_] ECL and enhancement of [Ru(bpy)_3_]^2+^ ECL within the mixed system (reactions (12)–(14)).

12 [Ir(ppy)_3_]* + [Ru(bpy)_3_]^3+^ → [Ir(ppy)_3_]^+^ + [Ru(bpy)_3_]^2+^*

13 [Ir(ppy)_3_]^–^ + [Ru(bpy)_3_]^2+^ → [Ir(ppy)_3_] + [Ru(bpy)_3_]^+^

14 [Ir(ppy)_3_]^–^ + [Ru(bpy)_3_]^3+^ → [Ir(ppy)_3_] + [Ru(bpy)_3_]^2+^*

Reaction (12) is analogous to that postulated by Moon *et al.*^8^ (reaction (9)) to explain the absence of ECL from [Ir(df-ppy)_2_(bpy)]^+^ when combined with [Ru(bpy)_3_]^2+^. Interestingly, whilst this pathway is certainly feasible within our system (Fig. 5), an examination of the reduction potentials within the mixed annihilation ECL system for which it was originally proposed suggests that it is unlikely to explain the energy transfer observed in that case (Fig. S5†). Reaction (13) is analogous to reaction (8), proposed by Swanick *et al.*^9^ to explain the lack of ECL from the iridium component of the [Ru(dtb-bpy)_3_]^2+^–[Ir(ppy)_2_(CN)_2_]^–^ system and an unexpectedly large electrochemical current for the [Ir(ppy)_2_(CN)_2_]^–^ reduction. These electron transfers (reactions (13) and (9)) are energetically feasible in both systems (Fig. 5 and S6†). Based on this process, Swanick *et al.* concluded that the electronically excited [Ir(ppy)_2_(CN)_2_]^–^* was not formed in their system.^9^ However, it is also possible that some of this excited state species is formed but then effectively quenched *via* electron exchange and resonance energy transfer, analogous to those discussed above. Moreover, the significant ion pairing interactions^17^ of the soft salt facilitate efficient quenching within that mixed annihilation ECL system.

### An additional route for enhancement in mixed annihilation ECL systems

The above discussion focuses on energy transfer between concomitant ECL systems under conditions that would be suitable to attain the excited state of either metal complex in isolation (*i.e.*, the applied potentials are generally beyond the first reduction and oxidation of both complexes). Under these conditions, considerable enhancement of the ECL of one complex in the mixture has been observed (compared to the annihilation ECL of that complex in isolation).^8^,^9^

**Fig. 6:**
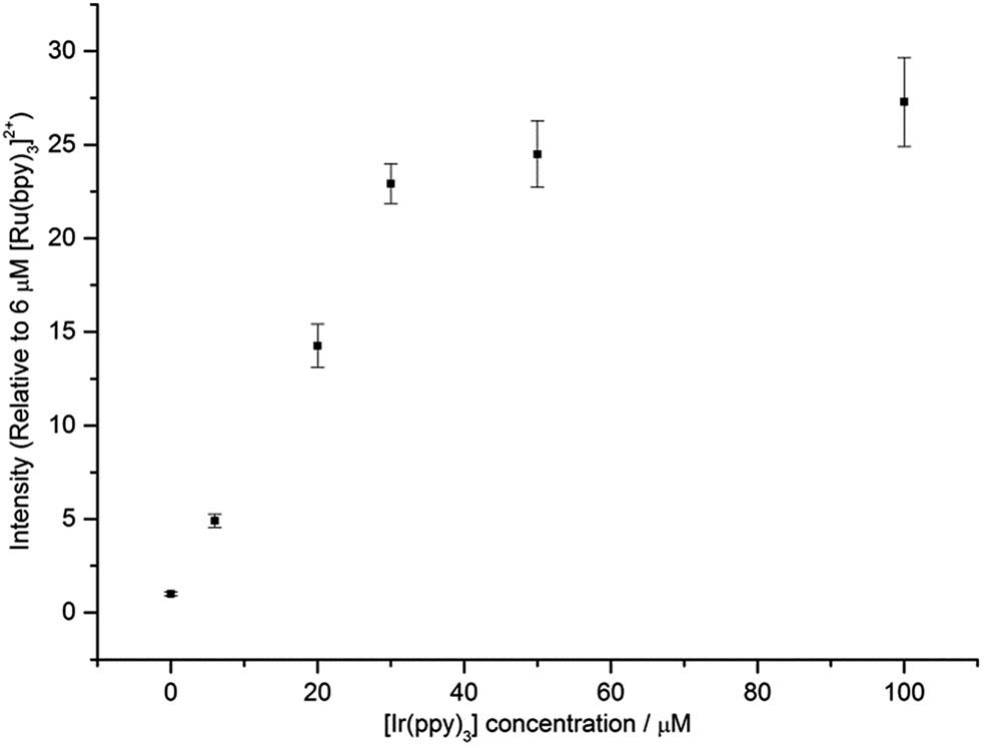
ECL intensity of [Ru(bpy)_3_]^2+^ (6 μM) in the presence of different concentrations of [Ir(ppy)_3_]. In each case, a two-step potential pulse was applied at 0.5 Hz for 12 s. Applied potentials: 0.99 V and –1.82 V *vs.* Fc^0/+^ (*i.e.*, 0.1 V beyond the oxidation and first reduction potential of [Ru(bpy)_3_]^2+^, respectively). All complexes were prepared in acetonitrile with 0.1 M [TBA][PF_6_]. Average RSD: 7.5%. In all cases, only the emission from the ruthenium complex was observed. The ECL spectrum for a mixture of 6 μM [Ru(bpy)_3_]^2+^ and 100 μM [Ir(ppy)_3_], under these applied potentials, is shown in Fig. S7.†

Fig. 6 shows that under conditions in which the generation of only one excited state is energetically feasible, we observed an additional mechanism of enhancement that does not involve the energy transfer pathways discussed above. In the [Ru(bpy)_3_]^2+^–[Ir(ppy)_3_] mixed annihilation ECL system for example, applying potentials of 0.99 V and –1.82 V (*vs.* Fc^0/+^) results in the formation of [Ru(bpy)_3_]^3+^, [Ir(ppy)_3_]^+^ and [Ru(bpy)_3_]^+^, but not [Ir(ppy)_3_]^–^ (Fig. 1). In this case, the [Ir(ppy)_3_]* emitter is not formed (Fig. 2), because there is insufficient free energy in the mixed annihilation ECL reaction of [Ir(ppy)_3_]^+^ and [Ru(bpy)_3_]^+^ (Δ*G*_es_ ∼ +0.41 eV, based on the data shown in Fig. 5). In contrast, the electronically excited [Ru(bpy)_3_]^2+^* species is generated by the reaction of [Ru(bpy)_3_]^+^ with not only [Ru(bpy)_3_]^3+^ (Δ*G*_es_ ∼ –0.48 eV), but also [Ir(ppy)_3_]^+^ (reaction (5) above; Δ*G*_es_ ∼ –0.04 eV). As the concentration of [Ir(ppy)_3_] in the mixture is increased, so too is the concentration of [Ir(ppy)_3_]^+^ when 0.99 V is applied, which increases the probability that [Ru(bpy)_3_]^+^ species (generated at the applied potential of –1.82 V) is oxidised to form [Ru(bpy)_3_]^2+^*, thus increasing the observed ECL from the ruthenium complex emitter (Fig. 6). An approximately linear increase (*R*^2^ = 0.996) in the annihilation ECL (integrated peak area) of 6 μM [Ru(bpy)_3_]^2+^ was observed with [Ir(ppy)_3_] concentration up to 30 μM. Beyond this point, the ECL intensity was approximately 25-fold greater than that of the annihilation ECL of [Ru(bpy)_3_]^2+^ in the absence of the iridium complex. It should be noted that the linear range and relative intensities were found to vary depending on the timespan of the applied potential, the presence of trace amounts of oxygen prior to analysis and relative concentrations of [Ru(bpy)_3_]^2+^ and [Ir(ppy)_3_]. Moreover, emission was sometimes also observed at the counter electrode^23^ and therefore we employed a collimating lens (focussed on the working electrode) to eliminate interference. ECL spectra were obtained to confirm that only the characteristic orange emission from [Ru(bpy)_3_]^2+^* was generated in this system using these applied potentials (Fig. S7†).

We also examined the enhancing effect of a non-emissive iridium complex, [Ir(ppz)_3_], on the annihilation ECL of [Ru(bpy)_3_]^2+^. The oxidation potential of [Ir(ppz)_3_] (0.38 V *vs.* Fc^0/+^; Fig. S8†) is slightly higher than that of [Ir(ppy)_3_], but unlike [Ir(ppy)_3_], [Ir(ppz)_3_] has a luminescence quantum yield below 0.01 at room temperature due to efficient population of a non-emissive metal-centred excited state.^24^ Applying potentials of –1.82 V and 0.99 V to a mixture of 6 μM [Ru(bpy)_3_]^2+^ and 60 μM [Ir(ppz)_3_] to generate [Ru(bpy)_3_]^+^, [Ru(bpy)_3_]^3+^ and [Ir(ppz)_3_]^+^, gave 59-fold (±2) ECL (integrated peak area) from [Ru(bpy)_3_]^2+^*, compared to that generated from [Ru(bpy)_3_]^2+^ in the absence of [Ir(ppz)_3_].

### A comparison of mixed metal-complex annihilation ECL systems

For a quantitative comparison of the three previously reported mixed metal-complex annihilation ECL systems, we examined the emission spectra of the Ru complex at a series of different concentrations (0.005 mM to 0.12 mM) in the presence and absence of the respective Ir complex (at 0.12 mM). We also tested the Ir complex (0.12 mM) in the absence of the Ru complex. For these experiments, a higher frequency electrochemical pulse (10 Hz) was used over the same acquisition time to enhance the ECL intensities.

For each system, potentials were selected to include both the first oxidation of each complex and the first reduction of each complex. In the [Ru(bpy)_3_]^2+^–[Ir(ppy)_3_] and [Ru(dtb-bpy)_3_]^2+^–[Ir(ppy)_2_(CN)_2_]^–^ systems (Fig. 5, S6a, S9 and S10†), pulsing 0.1 V beyond the first reduction of the Ir complex will also reach the 2^nd^, 3^rd^ and to some extent the 4^th^ reduction of the Ru complex. Whereas, in the [Ru(bpy)_3_]^2+^–[Ir(df-ppy)_2_(bpy)]^+^ system (Fig. S5a†), the first reduction potentials of the Ru and Ir complexes are similar.

**Fig. 7:**
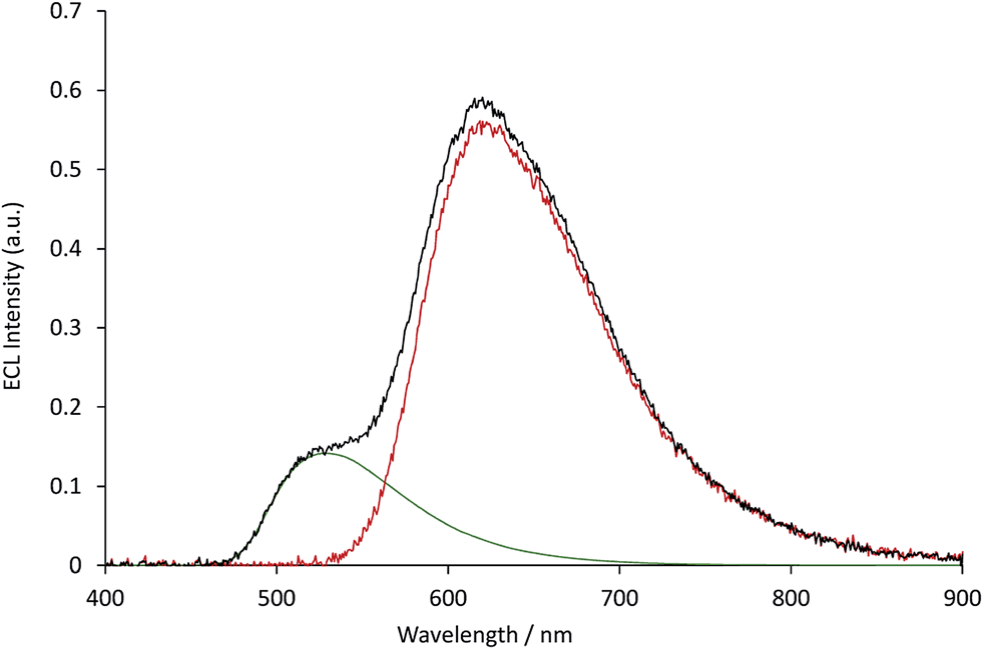
The deconvolution of the annihilation ECL spectrum (black plot) from 0.03 mM [Ru(bpy)_3_](PF_6_)_2_ and 0.12 mM [Ir(ppy)_3_] into the characteristic spectra of the two metal complexes (green and red plots, for which the spectral distributions were derived from the ECL of the individual complexes at 0.12 mM), using the Solver function of Microsoft Excel software. The ECL was generated using a two-step potential pulse (0.99 V and –2.77 V *vs.* Fc^0/+^) applied at 10 Hz for 12 s. Complexes were prepared in acetonitrile containing 0.1 M [TBA][PF_6_]. Additional examples are shown in Fig. S12.†

**Fig. 8:**
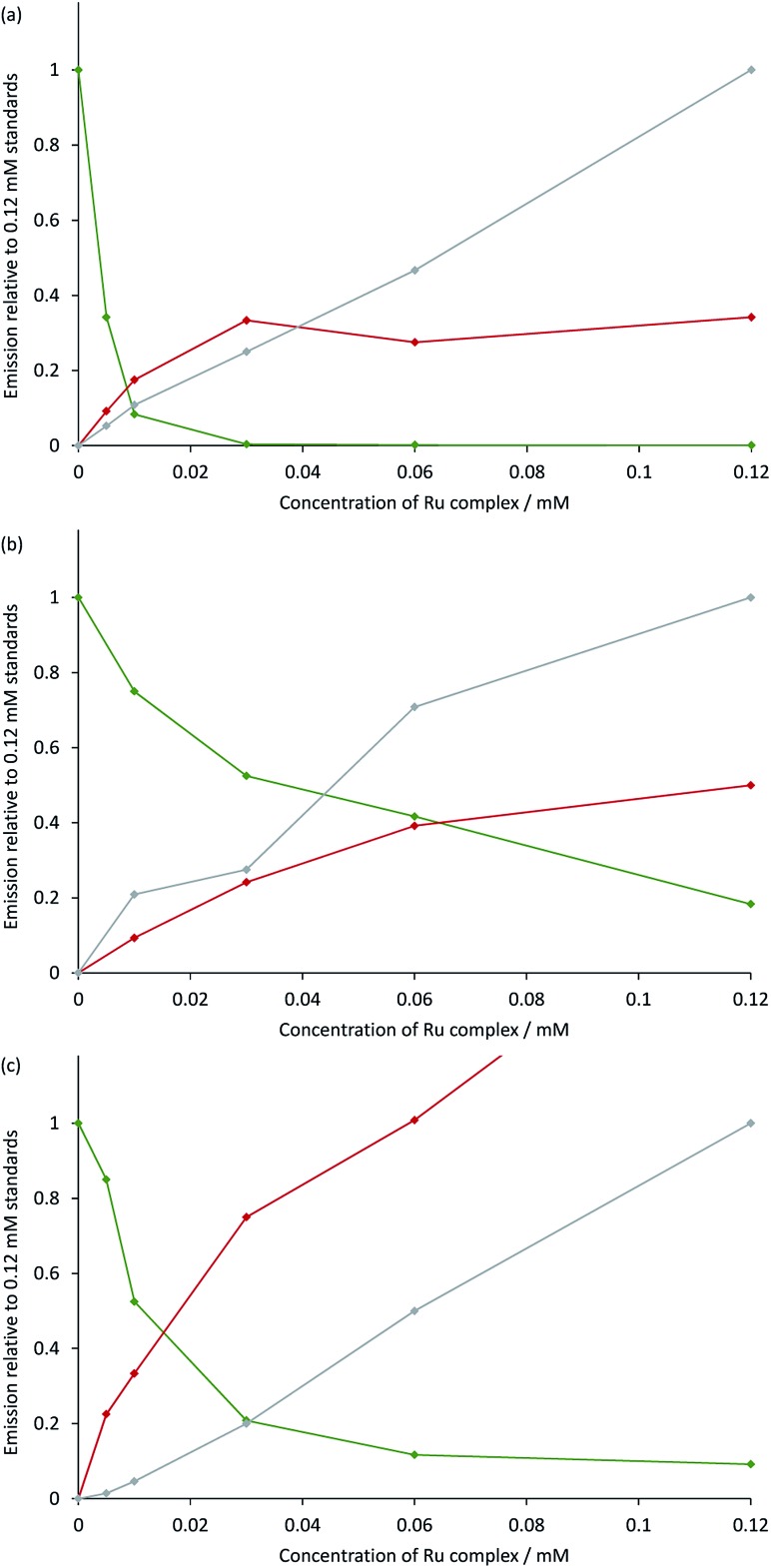
Annihilation ECL intensities from: (a) [Ru(bpy)_3_](PF_6_)_2_ and [Ir(ppy)_3_]; (b) [Ru(dtb-bpy)_3_](PF_6_)_2_ and TBA[Ir(ppy)_2_(CN)_2_]; or (c) [Ru(bpy)_3_](PF_6_)_2_ and [Ir(df-ppy)_2_(bpy)](PF_6_), in acetonitrile containing 0.1 M [TBA][PF_6_]. The green plot is the ECL intensity of the Ir complex in the mixed solutions, relative to that of an individual standard of the Ir complex (0.12 mM) in the absence of the Ru complex. The red and grey plots are the ECL intensities of the Ru complex (from 0 mM to 0.12 mM) with and without the presence of 0.12 mM Ir complex, respectively, relative to that of an individual standard of the Ru complex (0.12 mM). The absolute ECL intensities are shown in Fig. S13.† In each case, a two-step potential pulse was applied at 10 Hz for 12 s. The applied potentials were: (a) 0.99 V, –2.77 V *vs.* Fc^0/+^, (b) 0.83 V, –2.81 V *vs.* Fc^0/+^, (c) 1.20 V, –1.82 V *vs.* Fc^0/+^. ECL spectra from each mixed system were deconvoluted into their two characteristic components (Fig. S12†).

The measured ECL spectra (Fig. S11†) were deconvoluted into the two characteristic emission bands (defined by the ECL spectrum of each individual complex at 0.12 mM) using the Solver function of Excel (for examples, see Fig. 7 and S12†). This enabled not only the comparison of the absolute ECL intensities of the Ru and Ir complexes within each system without interference (Fig. S13†), but also their ECL intensities relative to that of a standard solution for each complex (Fig. 8).

Under these experimental conditions, we observed an efficient quenching of the annihilation ECL of [Ir(ppy)_3_] in the presence of increasing concentrations of [Ru(bpy)_3_]^2+^ (Fig. 8a). However, this did not translate to an enhancement of the ECL of the ruthenium complex at all concentrations. The grey and red plots in Fig. 8a show the ECL intensity of the [Ru(bpy)_3_]^2+^ complex at various concentrations in the absence and presence of [Ir(ppy)_3_], using the same applied potentials. At [Ru(bpy)_3_]^2+^ concentrations of 0.06 mM and 0.12 mM, the ECL intensities of both [Ir(ppy)_3_] and [Ru(bpy)_3_]^2+^ were below that of the corresponding individual complex. It is possible that this quenching involves electron transfer from [Ir(ppy)_3_] or from the reduced [Ir(ppy)_3_]^–^ to [Ru(bpy)_3_]^2+^*. Alternatively, the excitation pathway to the [Ru(bpy)_3_]^2+^* occurring *via* the concomitant iridium system may be less efficient than that of the direct annihilation of [Ru(bpy)_3_]^3+^ and [Ru(bpy)_3_]^+^.

We observed similar trends for the [Ru(dtb-bpy)_3_]^2+^–[Ir(ppy)_2_(CN)_2_]^–^ system (Fig. 8b), which is not surprising, considering the similarity of their redox potentials (Fig. S6†). The quenching of the iridium complex in this system was far less efficient (*K*_SV_ = 25; Fig. S14†) than that of [Ir(ppy)_3_] with [Ru(bpy)_3_]^2+^ (*K*_SV_ = 9.7 × 10^3^).

However, the ECL quantum yield of [Ir(ppy)_2_(CN)_2_]^–^ is far lower than that of [Ir(ppy)_3_], and the relative ECL intensities of the individual complexes at the same concentration (0.12 mM) under these conditions were found to increase in the order: [Ir(ppy)_2_(CN)_2_]^–^ ≪ [Ru(bpy)_3_]^2+^ < [Ru(dtb-bpy)_3_]^2+^ ≪ [Ir(ppy)_3_] (Fig. S13†). Therefore, although the iridium complex is quenched less efficiently in the [Ru(dtb-bpy)_3_]^2+^–[Ir(ppy)_2_(CN)_2_]^–^ system, its contribution to the overall ECL emission is lower than that of the iridium complex of the [Ru(bpy)_3_]^2+^–[Ir(ppy)_3_] system (compare, for example, Fig. S12b and S12d†). A very minor contribution to the overall ECL emission was observed from the iridium luminophore in the [Ru(dtb-bpy)_3_]^2+^–[Ir(ppy)_2_(CN)_2_]^–^ system at a 1 : 2 concentration ratio (Fig. S12h†), and to an ever lesser extent in the same stoichiometric ratio in the [Ru(dtb-bpy)_3_][Ir(ppy)_2_(CN)_2_]_2_ soft salt at 0.06 mM. In general agreement with the result of Swanick *et al.*,^9^ the ECL from these complexes at that concentration ratio arose almost entirely from the ruthenium component, but some [Ir(ppy)_2_(CN)_2_]^–^* was formed at all concentration ratios examined in our study. It is possible that a minor emission from [Ir(ppy)_2_(CN)_2_]^–^* was hidden in the noise of the ECL spectra obtained in that study,^9^ but it is also feasible that use of cyclic voltammetry to obtain ECL (rather than chronoamperometry) resulted in greater quenching of the iridium complex, and favoured the observed enhancement in ECL from the ruthenium complex.

In the case of the [Ru(bpy)_3_]^2+^–[Ir(df-ppy)_2_(bpy)]^+^ system (Fig. 8c), the quenching of the ECL from the iridium complex (*K*_SV_ = 125) was more efficient that observed in [Ru(dtb-bpy)_3_]^2+^–[Ir(ppy)_2_(CN)_2_]^–^ system, but still far less efficient than that of the [Ru(bpy)_3_]^2+^–[Ir(ppy)_3_] system. Moon *et al.*^8^ observed emission from only the ruthenium complex of the [Ru(bpy)_3_]^2+^–[Ir(df-ppy)_2_(bpy)]^+^ system, even at a stoichiometric ratio of 1 : 4. Under our conditions, we observed an emission from both complexes at that ratio (Fig. S12f†) although as the concentration of the ruthenium complex was increased to a stoichiometric ratio of 1 : 1, the contribution from the iridium complex decreased to less than 5% of the integrated ECL spectrum. The mixed system produced considerably greater ECL from [Ru(bpy)_3_]^2+^ than that of the individual complex. At the same [Ir(ppy)_2_(CN)_2_]^–^ concentration (0.12 mM), the degree of enhancement decreased with increasing concentration of [Ru(bpy)_3_]^2+^ (Fig. 8c and S15†). At stoichiometric ratios of 1 : 2 and 1 : 1, the enhancement was 2.0-fold and 1.7-fold, respectively, which was in reasonable agreement with the approximately 2-fold enhancement for a 3 : 2 mixture reported by Moon and co-workers.^8^

## Conclusions

The apparent differences in emission properties (*i.e.*, luminescence observed from a single luminophore or a combination of two luminophores) in the preliminary explorations of annihilation ECL of mixed metal complexes can largely be ascribed to the relative concentrations of the two complexes used in the respective studies, a variable that until now has been largely overlooked. Other important factors include the relative ECL quantum yields (or relative intensities) of the individual and mixed annihilation ECL reactions, and the efficiency of various energy transfer pathways. In two previous publications, the observed energy transfer was tentatively attributed to a specific electron-transfer reaction, but it is likely to arise from a combination of several concomitant pathways that may include resonance energy transfer or electron transfer/exchange between the numerous oxidised, reduced, ground and/or excited states of the complexes within the mixed annihilation ECL system. The feasibility and relative efficiency of these pathways are dependent on the inherent electrochemical and photophysical characteristics of the metal complexes and the applied electrode potentials, which will need to be carefully considered to create annihilation ECL systems containing near-equimolar mixtures of metal complexes that are capable of simultaneous emissions from two distinct luminophores.

## Supplementary Material

Supplementary information
