# Supplementary material for: New perspectives on the annihilation electrogenerated chemiluminescence of mixed metal complexes in solution
Source: Chem Sci. 2016 Apr 29;7(8):5271–9. doi: 10.1039/c6sc01570k (PMC6020550; doi:10.1039/c6sc01570k)
Supplement: Supplementary file 1 [file SC-007-C6SC01570K-s001.pdf]

Electronic Supplementary Material (ESI) for Chemical Science.
This journal is © The Royal Society of Chemistry 2016

Electronic Supplementary Information:

New Perspectives on the Annihilation Electrogenerated Chemiluminescence of Mixed Metal Complexes in Solution

Emily Kerr, Egan H. Doeven,* Gregory J. Barbante, Conor F. Hogan, David J. Hayne,

Paul S. Donnelly and Paul S. Francis*

Energetics of annihilation ECL

A detailed account of this topic can be found within the excellent explanation of "Marcus theory in the qualitative and quantitative description of electrochemiluminescence phenomena" by Andrzej Kapturkiewicz (*Adv. Electrochem. Sci. Eng.*, 1997, **5**, 1-60). A brief summary adapted to our description of the annihilation ECL of mixed metal complex systems is presented below:

The Gibbs free energy of the annihilation reaction to form ground electronic state products (reaction 3c in our paper) can be calculated as:

$$\Delta G_0 = E^\circ_{D+/D} - E^\circ_{A/A-} - w_r + w_p$$

where w_r is the energy required to bring the reactants together to the most probable separation distance at which the electron transfer takes place, and w_p is the energy required to bring the product into the precursor complex.

Similarly the Gibbs free energy for the formation of products with one in an electronically excited state (reactions 3a and 3b in our paper) can be calculated as:

$$\Delta G_0 = E^\circ_{D+/D} - E^\circ_{A/A-} - w_r + w_p + E_{es}(D^*)$$

$$\Delta G_0 = E^\circ_{D+/D} - E^\circ_{A/A-} - w_r + w_p + E_{es}(A^*)$$

These equations are not strictly correct because of the combination of Gibbs energy and energy terms. To overcome this inconsistency, the entropic contribution ΔS can be calculated as follows:

$$\Delta S = -\partial \Delta G_0/\partial T = -\partial[(E^\circ_{D+/D} - E^\circ_{A/A-}) - w_r + w_p]\ \partial T$$

However, only small values for ΔS (compared than those for the isolated ions) are generally obtained, due to the correction for the Coulombic interaction energy terms, and at least in the first approximation, the entropic contribution can be neglected.

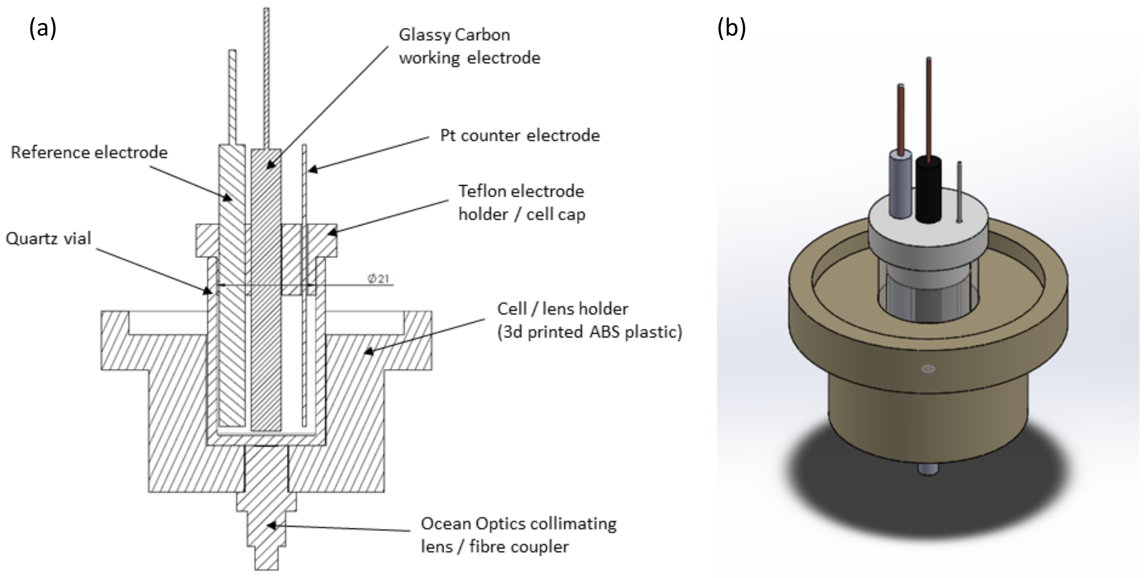

Figure S1. (a) Sectioned view and (b) 3D depiction of the custom built cell holder for ECL.

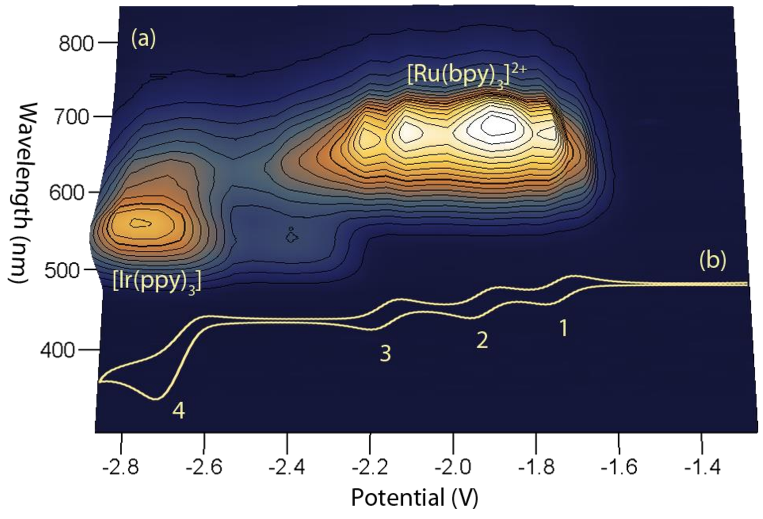

Figure S2. (a) A 3D representation of the ECL of the $[Ru(bpy)_3]^{2+}$-$[Ir(ppy)_3]$ mixed annihilation system spectra showing ECL intensity versus emission wavelength and applied reductive potential (V *vs* $Fc^{0/+}$). Data were obtained using an automated chronoamperometry procedure with an oxidative potential of 0.43 V and a series of reductive potentials spaced 50 mV apart, using 0.01 mM $[Ru(bpy)_3]^{2+}$ and 0.24 mM $[Ir(ppy)_3]$ in acetonitrile with 0.1 M $[TBA][PF_6]$. (b) The corresponding portion of a cyclic voltammogram of 0.25 mM $[Ru(bpy)_3]^{2+}$ and 0.25 mM $[Ir(ppy)_3]$ (0.1 M $[TBA][PF_6]$, acetonitrile), showing: (1) $[Ru(bpy)_3]^{1+/2+}$; (2) $[Ru(bpy)_3]^{0/1+}$; (3) $[Ru(bpy)_3]^{1-/0}$; and (4) a combination of $[Ir(ppy)_3]^{1-/0}$ and $[Ru(bpy)_3]^{2-/1-}$.

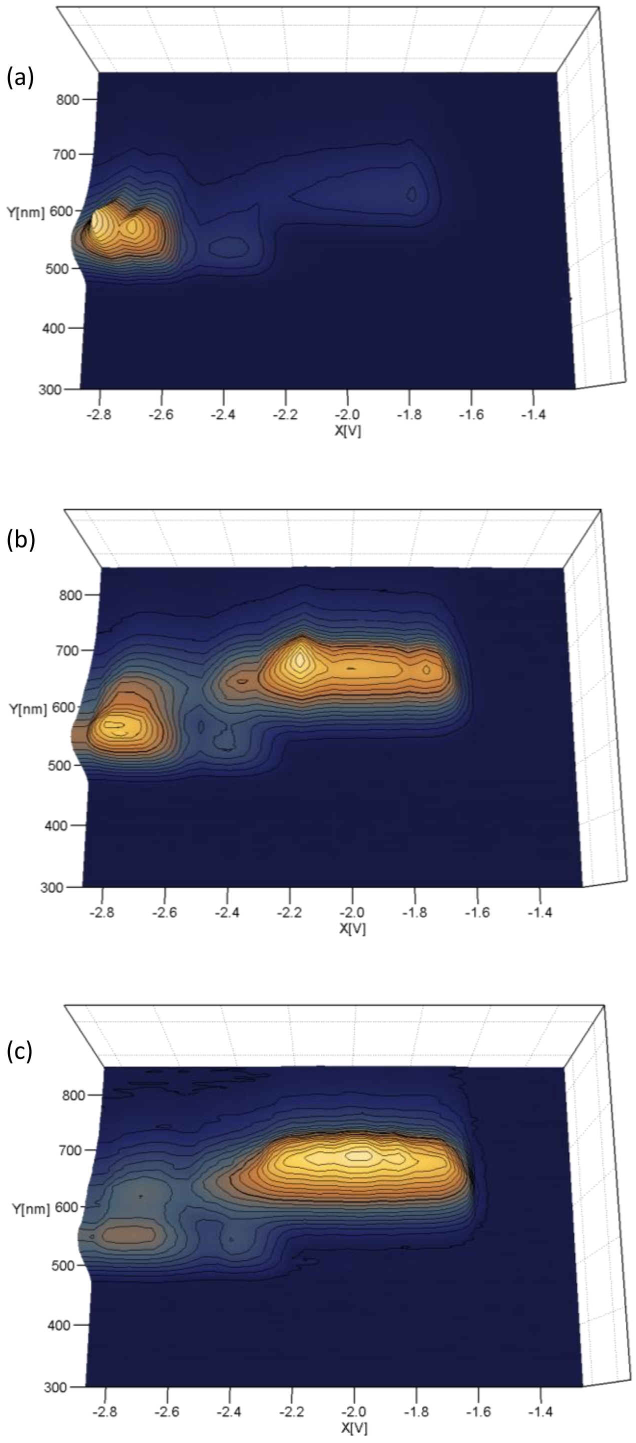

Figure S3 (a) 3D representations (normalised ECL intensity versus emission wavelength and applied reductive potential) for the $[Ru(bpy)_3]^{2+}$-$[Ir(ppy)_3]$ mixed annihilation system using three different concentrations of $[Ru(bpy)_3]^{2+}$: (a) 0.005 mM, (b) 0.010 mM, and (c) 0.015 mM, where the X axis is potential (V *vs* $Fc^{0/+}$) and Y axis is wavelength (nm). Each data set was obtained using 0.24 mM $[Ir(ppy)_3]$, with an oxidative potential of 0.99 V and a series of reductive potentials spaced 50 mV apart. Complexes were prepared in acetonitrile with 0.1 M $[TBA][PF_6]$.

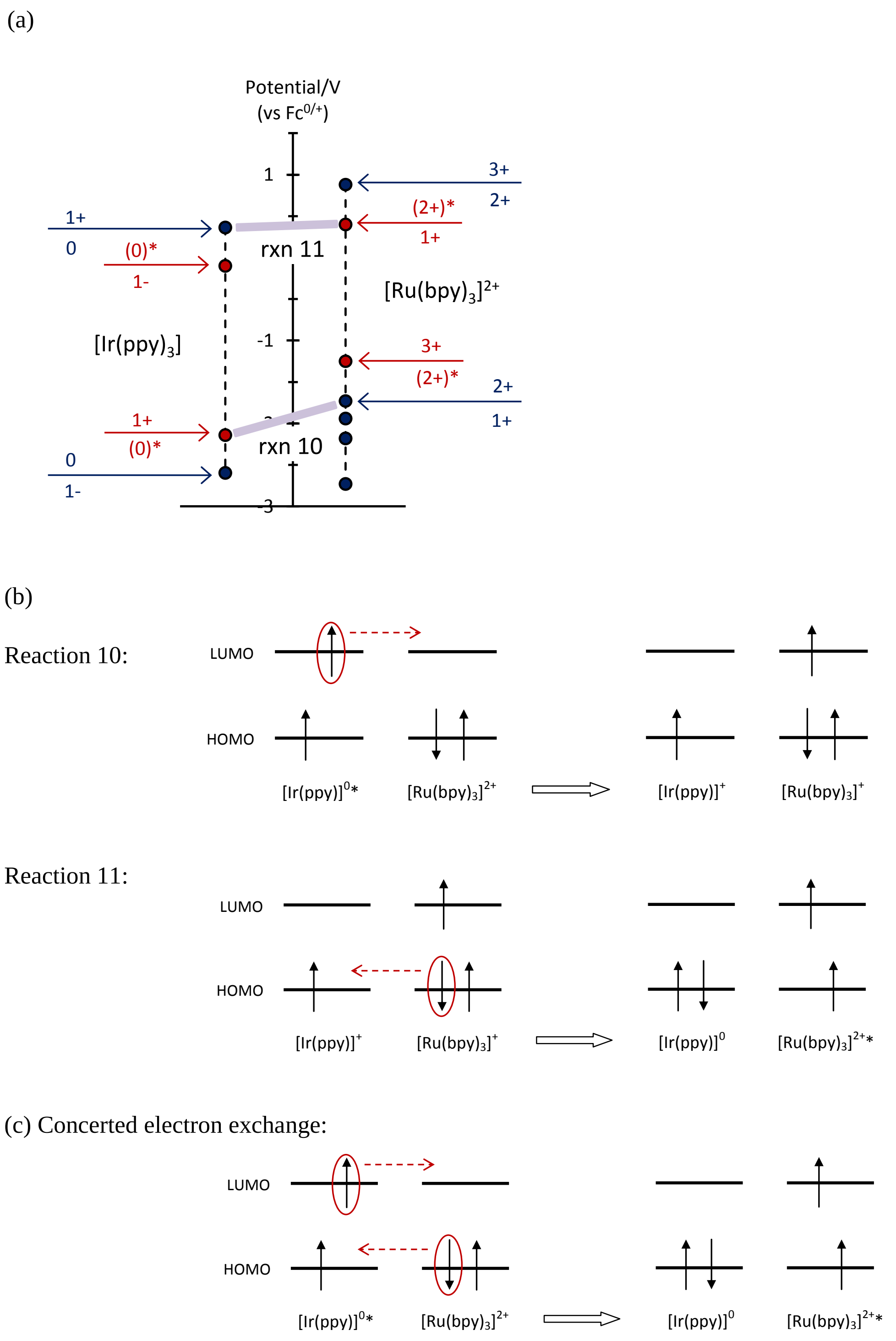

Figure S4. (a) Electron transfer between the ground and excited states of the most stable oxidation state complexes in the $[Ru(bpy)_3]^{2+}$-$[Ir(ppy)_3]$ mixed annihilation ECL system; (b) Generalised depiction of stepwise process; and (c) Concerted electron exchange.

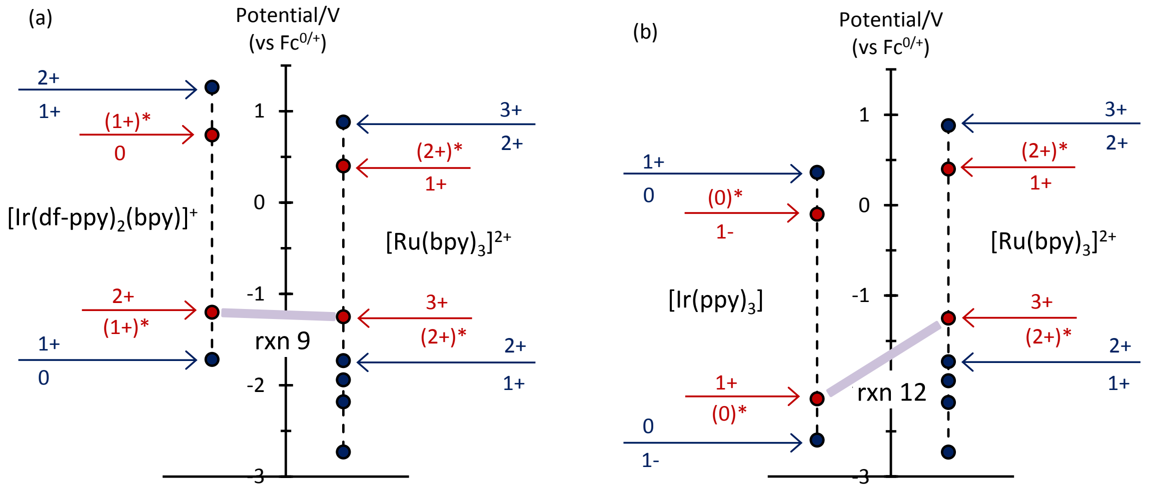

Figure S5. (a) Redox potentials for ground and excited states within the $[Ru(bpy)_3]^{2+}$-$[Ir(df\text{-}ppy)_2(bpy)]^{+}$ system examined by Moon and co-workers (J. Am. Chem. Soc., 2014, **136**, 3705-3712), showing the reaction proposed by the authors to account for the absence of ECL from the iridium-complex component. (b) The analogous electron transfer in the $[Ru(bpy)_3]^{2+}$-$[Ir(ppy)_3]$ system.

Data for Figure S5(a): Moon *et al.*[1] reported the electrochemical potentials of the $[Ru(bpy)_3]^{2+}$ complex in acetonitrile as 0.91 V and -1.24 V *vs* $Fc^{0/+}$. The oxidative potential is in reasonable agreement with previous data, but the reductive potential is significantly different (*c.f.* Figure 1 in main text),[2] which appears to be due to an uncharacteristic artifact within their cyclic voltammogram[1] for that complex. For Figure S5(a), we used the potentials for the ground and ^{3}MLCT excited state of $[Ru(bpy)_3]^{2+}$ reported by Juris *et al.*[2] (ground state: 1.26 V and -1.35 V; excited state: -0.87 V and 0.78 V *vs* SCE), referenced to the ferrocene/ferrocenium couple using the conversion constant of -380 mV in acetonitrile at 25ºC,[3] to give: -1.25 V and 0.40 V *vs* $Fc^{0/+}$ (excited state); 0.88 V, -1.73 V *vs* $Fc^{0/+}$ (ground state), which are in good agreement with our data. The subsequent reduction potentials were from our data (Figure 1).

The potentials reported by Moon *et al.*[1] for the $[Ir(df\text{-}ppy)_2(bpy)]^{-}$ complex (1.23 V and -1.34 V *vs* $Fc^{0/+}$) in acetonitrile are also questionable, because the difference between these potentials of 2.57 V is unexpectedly low for an iridium complex exhibiting green luminescence. Unfortunately, the previously reported potentials for $[Ir(df\text{-}ppy)_2(bpy)]^{-}$ are inconsistent.[1, 4, 5] For Figure S5(a), we used the data reported by Singh and co-workers,[5] which included an estimation of the potentials of the excited state complex. Although Singh *et al.*[5] stated that their potentials (obtained in acetonitrile) were reported relative to the ferrocene/ferrocenium redox couple, this does not appear to be the case, considering the values presented for well-known complexes such as $[Ir(ppy)_3]$. After correction (by -0.40 V) to account for this, we obtain 1.26 V and -1.72 V *vs* $Fc^{0/+}$ (ground); -1.20 V and 0.74 V *vs* $Fc^{0/+}$ (excited). The ground state oxidative potential 1.26 V *vs* $Fc^{0/+}$ is similar to that reported by Moon *et al.*[1] (1.23 V *vs* $Fc^{0/+}$) and the reductive potential (-1.72 V *vs* $Fc^{0/+}$) is visually coincident with the second reduction peak on the cyclic voltammogram presented by Moon *et al.*[1] for that complex.

To confirm our selection of this data, we synthesised and characterised the $[Ir(df\text{-}ppy)_2(bpy)](PF_6)$ complex (details on page S16). Our cyclic voltammetry (E_{ox} = 1.20 V *vs* $Fc^{0/+}$, E_{red} = -1.72 V *vs* $Fc^{0/+}$; Fig. S9a) was in reasonable agreement with that of Singh *et al.*,[5] after the correction noted above.

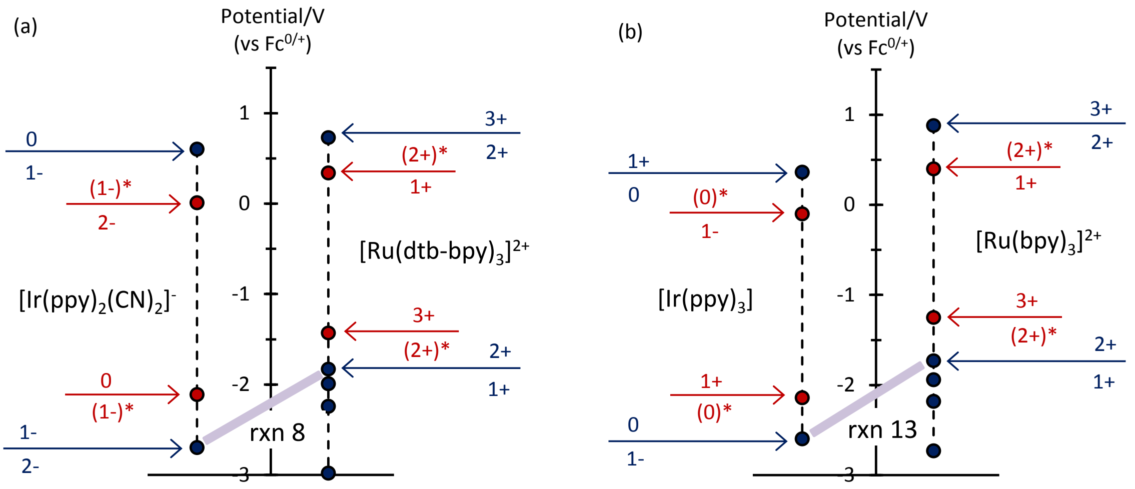

Figure S6. (a) Redox potentials for ground and excited states within the $[Ru(dtb\text{-}bpy)_3]^{2+}$-$[Ir(ppy)_2(CN)_2]^-$ system reported by Swanick and co-workers (Chem. Eur. J., 2015, 21, 7435-7440), showing the pathway for the electrocatalytic reduction of the ruthenium complex proposed by the authors to account for the absence of emission from the iridium component of the mixed system. (b) The analogous electron transfer in the $[Ru(bpy)_3]^{2+}$-$[Ir(ppy)_3]$ system.

Data for Figure S6(a): The electrochemical potentials for the ground state $[Ir(ppy)_2(CN)_2]^-$ complex were obtained from Swanick *et al.*[6] (0.98 V (*irreversible*) and -2.32 V *vs* SCE) and referenced to the ferrocene/ferrocenium couple using the conversion constant[3] of -380 mV in acetonitrile at 25°C (to give 0.60 V and -2.70 V *vs* $Fc^{0/+}$). The potentials for the excited-state $[Ir(ppy)_2(CN)_2]^-$* complex (-2.11 V and 0.01 V *vs* $Fc^{0/+}$) were estimated as the difference between the potentials of the ground state complex and the E^{00} energy of the low-temperature ^{3}MLCT emission, which was reported by Li *et al.*[7] as 458 nm (2.71 eV) in 2-methyltetrahydrofuran at 77 K, and by Chan *et al.*[8] as 457 nm (2.71 eV) in 4:1 (v/v) ethanol:methanol at 77 K. We note that the potentials obtained in acetonitrile by Swanick *et al.*[6] for the ground state $[Ir(ppy)_2(CN)_2]^-$ are similar to those obtained in dimethylformamide by Li *et al.*[7] (0.50 V (*irreversible*) and -2.78 V *vs* $Fc^{0/+}$) and in dichloromethane by Di Censo *et al.*[9] (0.55 V (*quasi-reversible*) and -2.69 V *vs* $Fc^{0/+}$).

The electrochemical potentials for the ground state $[Ru(dtb\text{-}bpy)_3]^{2+}$ complex were obtained from Swanick *et al.*[6] (1.11 V and -1.45 V *vs* SCE) and referenced to the ferrocene/ferrocenium couple using the conversion constant[3] of -380 mV in acetonitrile at 25°C, to give 0.73 V and -1.83 V *vs* $Fc^{0/+}$, which was in good agreement with previously published data.[2] The subsequent reduction potentials (-1.99 V, -2.24 V and -2.98 V *vs* $Fc^{0/+}$) were derived from the electrochemical data of the $[Ru(dtb\text{-}bpy)_3][Ir(ppy)_2(CN)_2]_2$ soft salt.[6] The excited-state potentials (-1.43 V and 0.34 V *vs* $Fc^{0/+}$) were estimated based on the low temperature E^{00} emission energy, which was reported by Juris *et al.*[2] to be 575 nm (2.16 eV) in 4:1 (v/v) methanol:ethanol at 77 K.

To confirm the oxidation and reduction potentials, we synthesised the $[Ru(dtb\text{-}bpy)_3](PF_6)_2$ and $TBA[Ir(ppy)_2(CN)_2]$ complexes (details on pages S16-S17). Our cyclic voltammetry (E_{ox} = 0.73 V, E_{red} = -1.83 V, -2.01 V, -2.30 V *vs* $Fc^{0/+}$ for $[Ru(dtb\text{-}bpy)_3](PF_6)_2$, and E_{ox} = 0.58 V, E_{red} = -2.71 V *vs* $Fc^{0/+}$ for $TBA[Ir(ppy)_2(CN)_2]$; Fig. S10a) was in good agreement with that reported by Swanick and co-workers.[6]

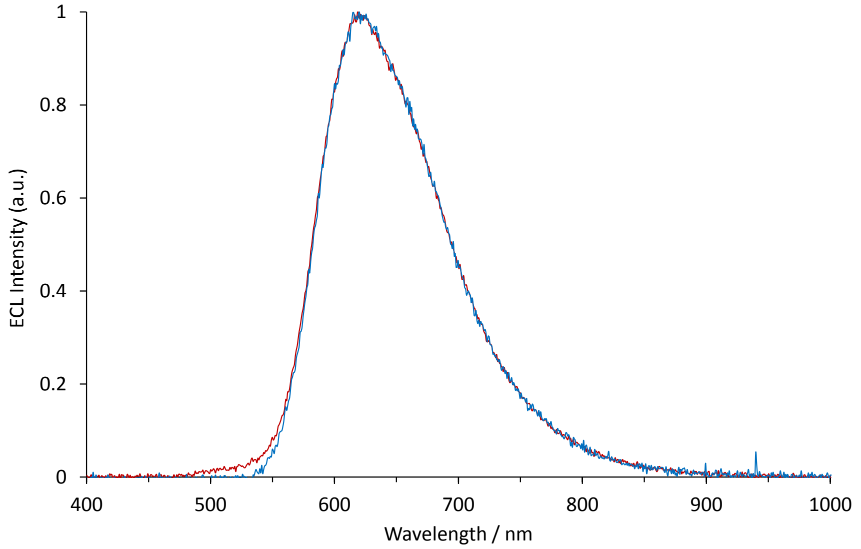

Figure S7. Normalised annihilation ECL spectra (applied potentials: 0.98 V and -1.82 V *vs* $Fc^{0/+}$) for a mixture of 0.006 mM $[Ru(bpy)_3]^{2+}$ and 0.100 mM $[Ir(ppy)_3]$ (red plot), and $[Ru(bpy)_3]^{2+}$ only (blue plot), in acetonitrile with 0.1 M $[TBA][PF_6]$. In this experiment the applied electrode potentials are 100 mV beyond the oxidation and first reduction of the $[Ru(bpy)_3]^{2+}$ complex. At 0.98 V, the $[Ir(ppy)_3]$ complex is oxidised, but at -1.83 V it is not reduced. As described in our previous work (Kerr *et al.*, Chemical Science, 2015, 6, 472-479), the annihilation ECL reaction between $[Ru(bpy)_3]^+$ and $[Ir(ppy)_3]^+$ is sufficiently energetic to form $[Ru(bpy)_3]^{2+}*$, but not $[Ir(ppy)_3]*$.

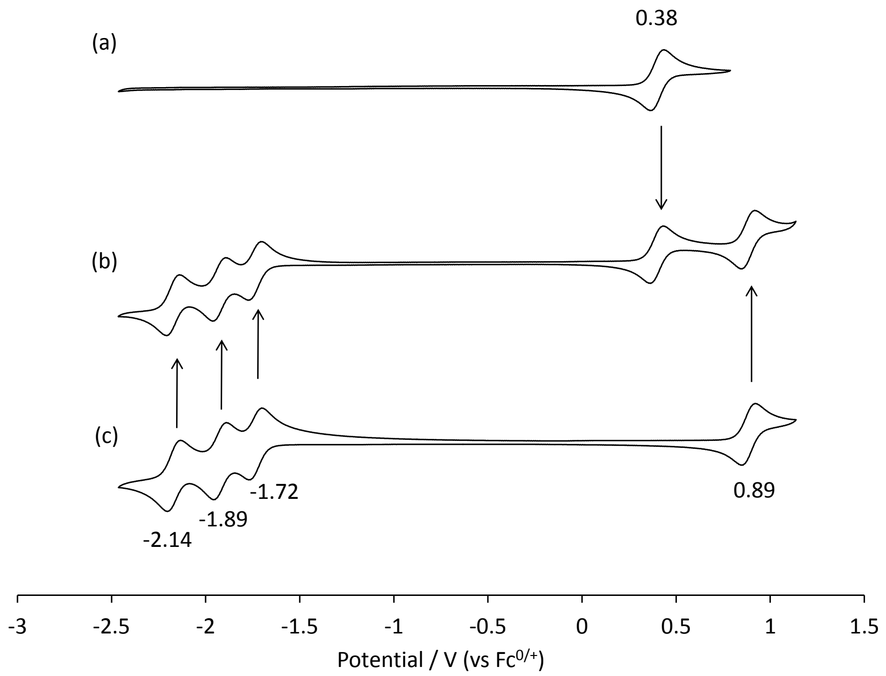

Figure S8. Cyclic voltammogram of (a) $[Ir(ppz)_3]$, (b) a mixture of $[Ir(ppz)_3]$ and $[Ru(bpy)_3]^{2+}$, and (c) $[Ru(bpy)_3]^{2+}$, showing E^0 values. Scan rate: 0.1 V s^{-1}. Complexes at 0.25 mM with 0.1 M $[TBA][PF_6]$ supporting electrolyte in acetonitrile.

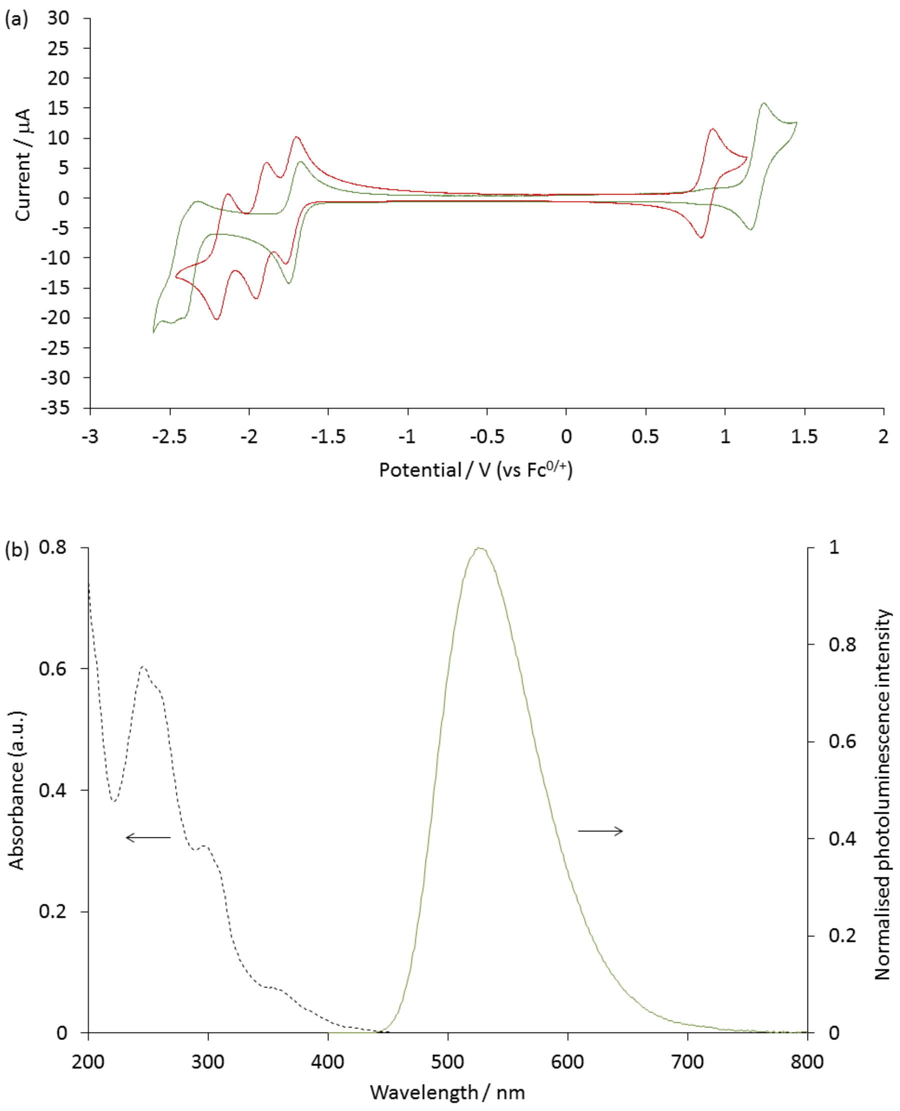

Figure S9. (a) Cyclic voltammograms of [Ir(df-ppy)$_2$(bpy)](PF$_6$) (green line) and [Ru(bpy)$_3$](PF$_6$)$_2$ (red line) at 0.5 mM with 0.1 M [TBA][PF$_6$] supporting electrolyte in acetonitrile. Scan rate: 0.1 V s^{-1}. Potentials for [Ir(df-ppy)$_2$(bpy)](PF$_6$): E_{ox} = 1.20 V *vs* Fc$^{0/+}$ and E_{red} = -1.72 V *vs* Fc$^{0/+}$. (b) Absorption spectrum (dashed black line) and corrected photoluminescence emission spectrum (green line) of [Ir(df-ppy)$_2$(bpy)](PF$_6$) at 10 μM. These metal complexes were used by Moon *et al*. (J. Am. Chem. Soc., 2014, **136**, 3705-3712) in their examination of the ECL of mixtures of metal complexes for flexible emissive displays.

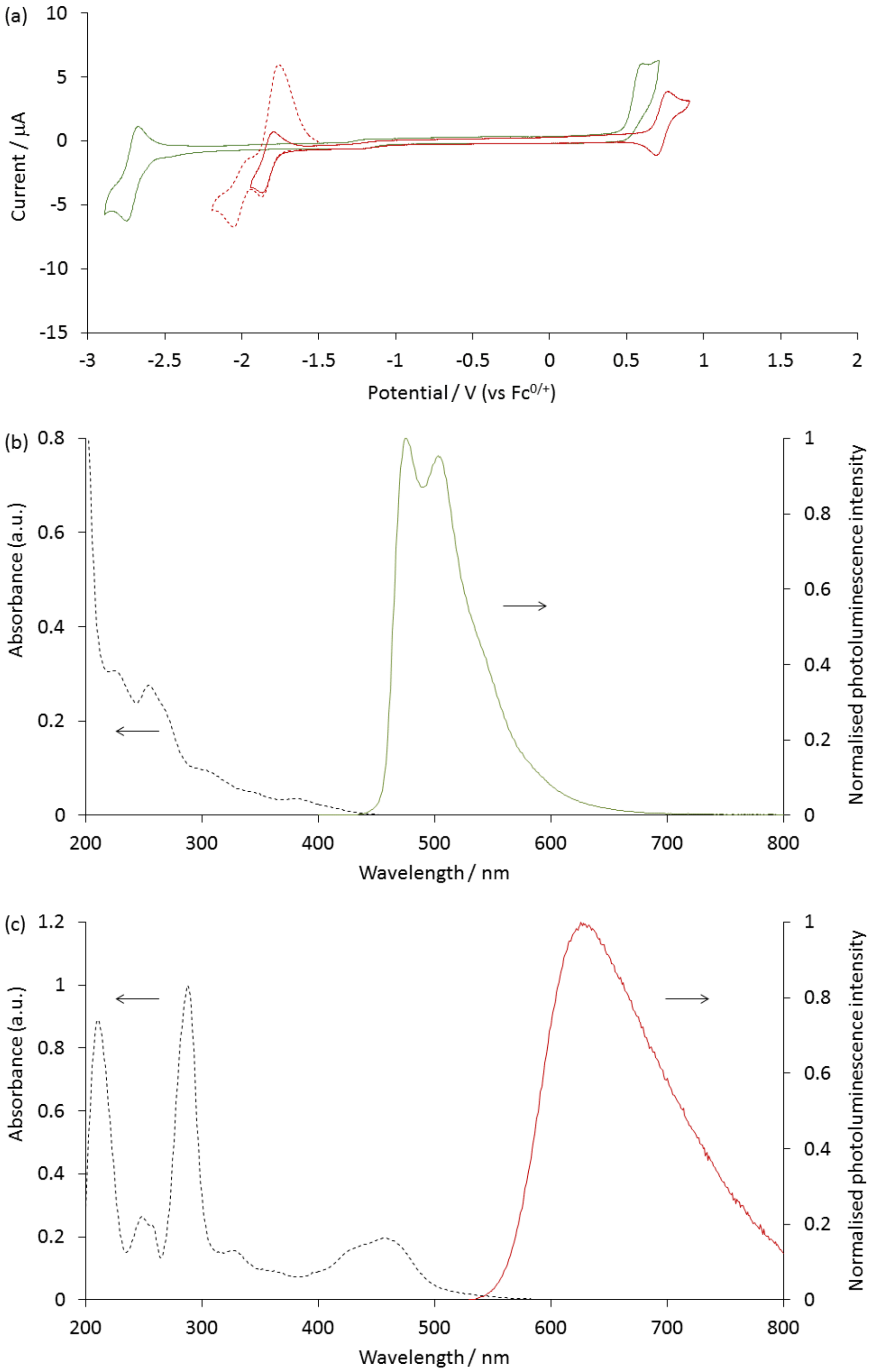

Figure S10. (a) Cyclic voltammograms of TBA[Ir(ppy)$_2$(CN)$_2$] (green line) and [Ru(dtb-bpy)$_3$](PF$_6$)$_2$ (red line) at 0.2 mM with 0.1 M [TBA][PF$_6$] in acetonitrile, scanning to the oxidation (E_{ox} = 0.58 V and 0.73 V *vs* Fc$^{0/+}$) and first reduction (E_{red} = -2.71 V and -1.83 V *vs* Fc$^{0/+}$) potentials. The 2nd and 3rd reductions of [Ru(dtb-bpy)$_3$](PF$_6$)$_2$ were at -2.01 V and -2.30 V *vs* Fc$^{0/+}$. When scanning beyond the first reduction of [Ru(dtb-bpy)$_3$](PF$_6$)$_2$ (e.g., dashed line), we observed a large peak at -1.76 V *vs* Fc$^{0/+}$ during the return scan. Scan rate: 0.1 V s^{-1}. (b) Absorption spectrum (dashed line) and corrected emission spectrum (green line) of TBA[Ir(ppy)$_2$(CN)$_2$] at 10 µM. (c) Absorption spectrum and corrected emission spectrum of [Ru(dtb-bpy)$_3$](PF$_6$)$_2$ at 10 µM. These complexes were previously examined by Swanick *et al*. (Chem. Eur. J., 2015, **21**, 7435-7440) in their study of the ECL of the [Ru(dtb-bpy)$_3$][Ir(ppy)$_2$(CN)$_2$]$_2$ soft salt.

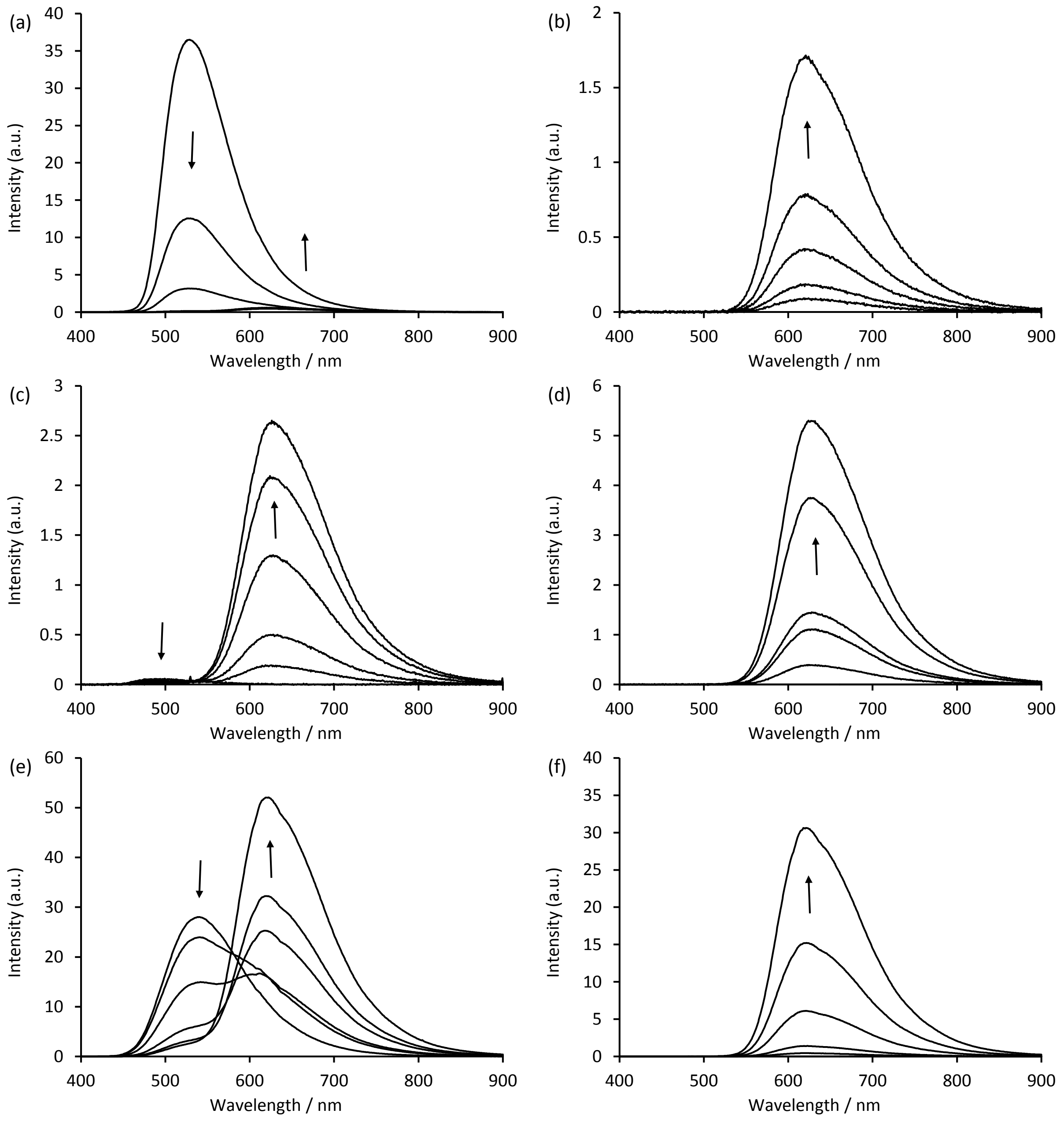

Figure S11. Annihilation ECL spectra from (a) 0 and 0.005-0.120 mM [Ru(bpy)$_3$](PF$_6$)$_2$ with 0.12 mM [Ir(ppy)$_3$], using 0.99 V and -2.77 V *vs* Fc$^{0/+}$; (b) 0.005-0.120 mM [Ru(bpy)$_3$](PF$_6$)$_2$, using 0.99 V and -2.77 V *vs* Fc$^{0/+}$; (c) 0 and 0.005-0.120 mM [Ru(dtb-bpy)$_3$](PF$_6$)$_2$ with 0.12 mM TBA[Ir(ppy)$_2$(CN)$_2$], using 0.83 V and -2.81 V *vs* Fc$^{0/+}$; (d) 0 and 0.005-0.120 mM [Ru(dtb-bpy)$_3$](PF$_6$)$_2$, using 0.83 V and -2.81 V *vs* Fc$^{0/+}$; (e) 0.005-0.120 mM [Ru(bpy)$_3$](PF$_6$)$_2$ with 0.12 [Ir(df-ppy)$_2$(bpy)](PF$_6$), using 1.20 V and -1.82 V *vs* Fc$^{0/+}$; (f) 0 and 0.005-0.120 mM [Ru(bpy)$_3$](PF$_6$)$_2$, using 1.20 V and -1.82 V *vs* Fc$^{0/+}$. In each case, complexes were prepared in acetonitrile containing 0.1 M [TBA][PF$_6$]. A two-step potential pulse was applied at 10 Hz for 12 s. The arrows show the change in emission intensity with increase in ruthenium complex concentration (after deconvolution procedure show in Fig. S12 was applied).

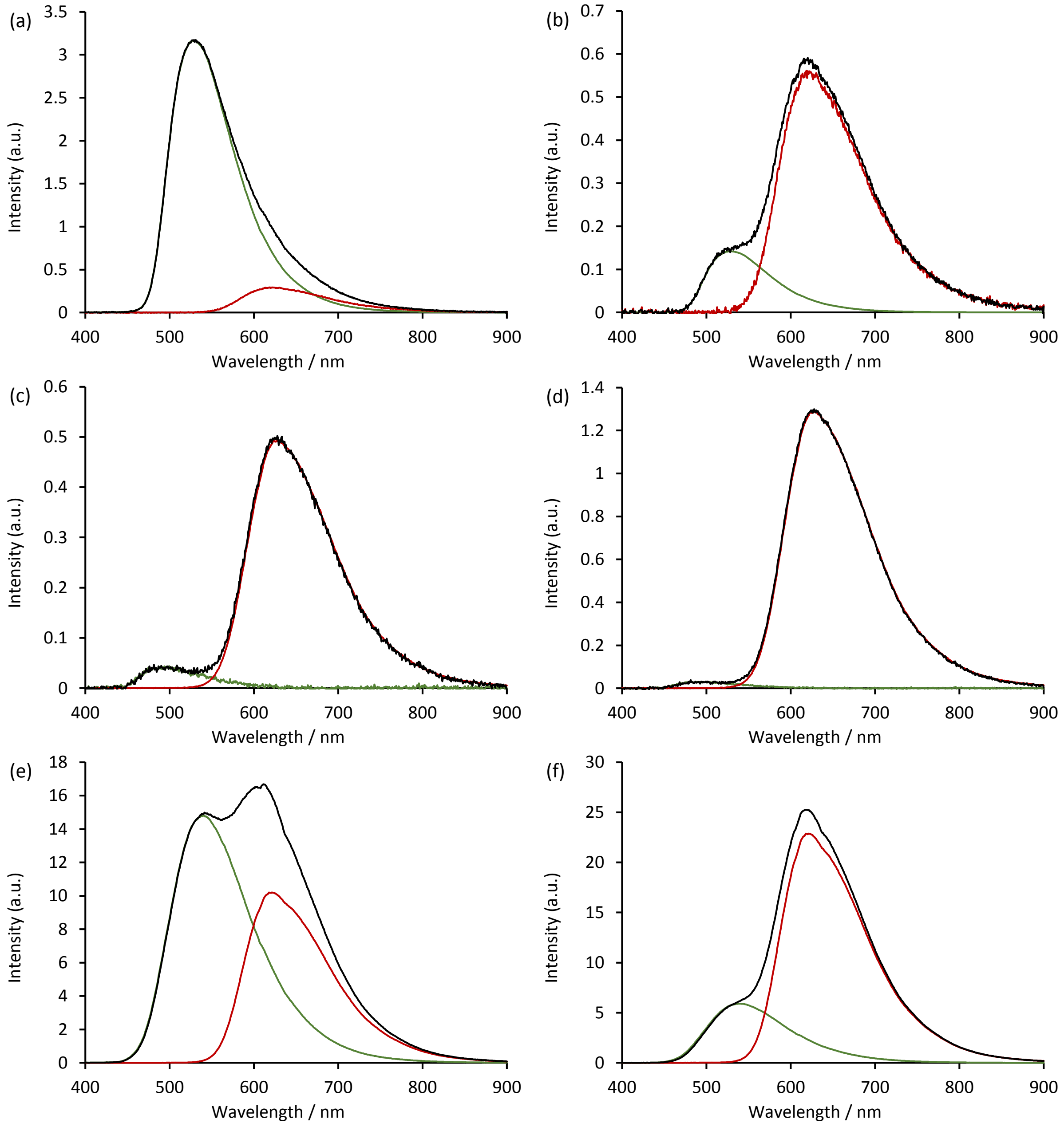

Figure S12. Examples of the deconvolution of the annihilation ECL spectra from mixed system into the characteristic spectra of the two individual metal complexes, using the Solver function of Microsoft Excel software. Complexes: (a) 0.01 mM $[Ru(bpy)_3](PF_6)_2$ and 0.12 mM $[Ir(ppy)_3]$; (b) 0.03 mM $[Ru(bpy)_3](PF_6)_2$ and 0.12 mM $[Ir(ppy)_3]$; (c) 0.01 mM $[Ru(dtb\text{-}bpy)_3](PF_6)_2$ and 0.12 mM $TBA[Ir(ppy)_2(CN)_2]$; (d) 0.03 mM $[Ru(dtb\text{-}bpy)_3](PF_6)_2$ and 0.12 mM $TBA[Ir(ppy)_2(CN)_2]$; (e) 0.01 mM $[Ru(bpy)_3](PF_6)_2$ and 0.12 $[Ir(df\text{-}ppy)_2(bpy)](PF_6)$; (f) 0.03 mM $[Ru(bpy)_3](PF_6)_2$ and 0.12 $[Ir(df\text{-}ppy)_2(bpy)](PF_6)$; in acetonitrile containing 0.1 M $[TBA][PF_6]$. In each case, the ECL was generated using a two-step potential pulse was applied at 10 Hz for 12 s. The applied potentials were: (a,b) 0.99 V and -2.77 V *vs* $Fc^{0/+}$; (c,d) (0.83 V and -2.81 V *vs* $Fc^{0/+}$; (e,f) 1.20 V and -1.82 V *vs* $Fc^{0/+}$.

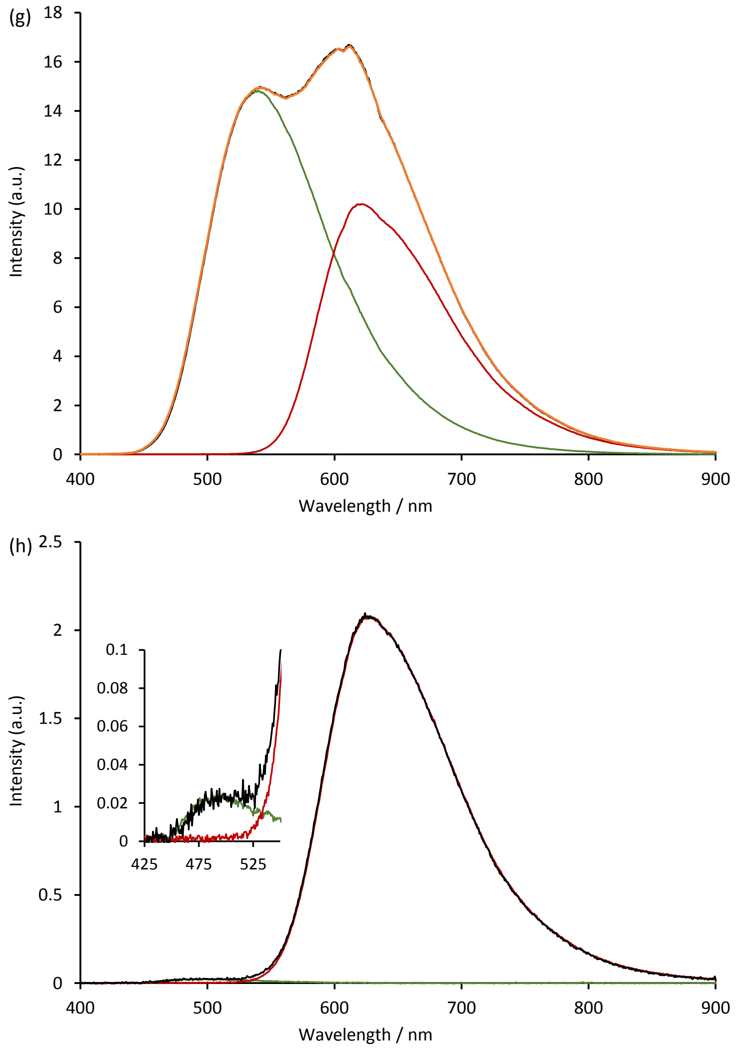

Figure S12 (continued). (g) A repeat of Fig. S12e showing the sum of the two individual components (orange plot) overlaid onto the original ECL spectrum (black plot). (h) Annihilation ECL spectrum and deconvoluted emissions for a mixture of 0.06 mM [Ru(dtb-bpy)$_3$](PF$_6$)$_2$ and 0.12 mM TBA[Ir(ppy)$_2$(CN)$_2$]. Inset: zoomed in section of the graph showing the emission from the Ir complex.

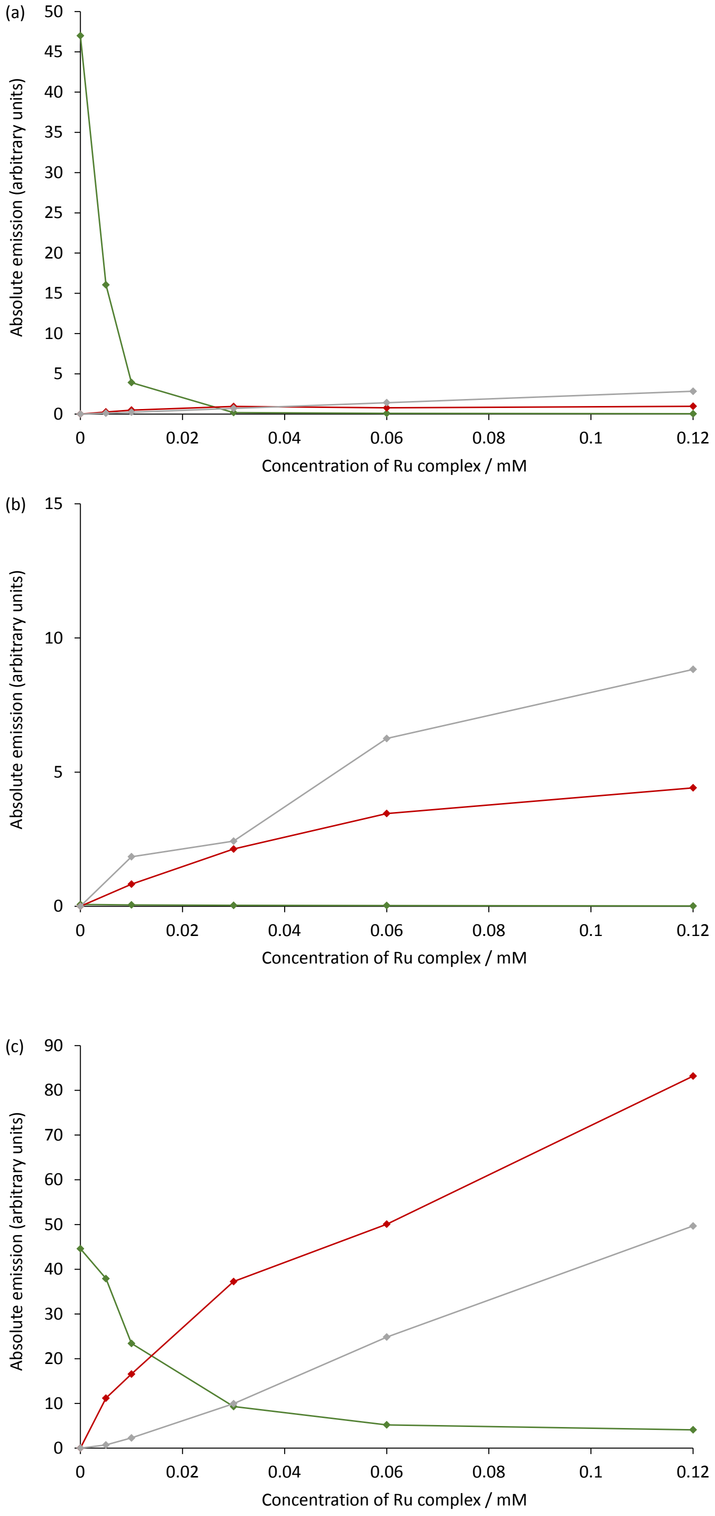

Figure S13. Annihilation ECL intensities from: (a) $[Ru(bpy)_3](PF_6)_2$ and $[Ir(ppy)_3]$; (b) $[Ru(dtb\text{-}bpy)_3](PF_6)_2$ and TBA$[Ir(ppy)_2(CN)_2]$; or (c) $[Ru(bpy)_3](PF_6)_2$ and $[Ir(df\text{-}ppy)_2(bpy)](PF_6)$, in acetonitrile containing 0.1 M [TBA]$[PF_6]$. The green plots are the ECL intensities of the Ir complex in the mixed solutions. The red and grey plots are the ECL intensities of the Ru complex (from 0 to 0.12 mM) with and without the presence of 0.12 mM Ir complex, respectively. In each case, a two-step potential pulse was applied at 10 Hz for 12 s. The applied potentials were: (a) 0.99 V and -2.77 V *vs* $Fc^{0/+}$, (b) (0.83 V and -2.81 V *vs* $Fc^{0/+}$, (c) 1.20 V and -1.82 V *vs* $Fc^{0/+}$. The ECL spectrum from each mixed solution was deconvoluted into its two characteristic components as shown in Fig. S12. The ECL intensities for the Ru and Ir complexes relative to standards of each individual complex are shown in Fig. 8 in the paper.

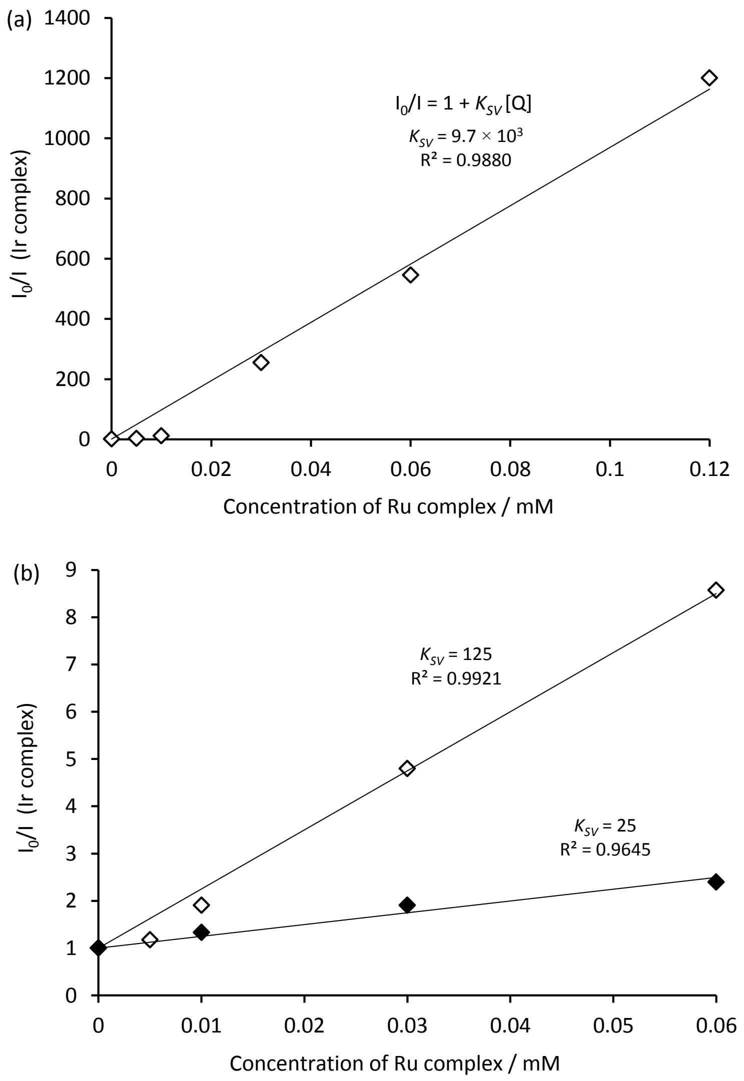

Figure S14. Quenching of the ECL of the Ir complex in solutions containing: (a) [Ir(ppy)$_3$] and [Ru(bpy)$_3$](PF$_6$)$_2$; (b) [Ir(df-ppy)$_2$(bpy)](PF$_6$) and [Ru(bpy)$_3$](PF$_6$)$_2$ (white dots), or TBA[Ir(ppy)$_2$(CN)$_2$] and [Ru(dtb-bpy)$_3$](PF$_6$)$_2$ (black dots). The ECL was generated by applying potentials of sufficient magnitude to alternately oxidise and reduce both complexes in solution, as described in Fig. S13. The deconvolution process (shown in Fig. S12) was applied to all ECL spectra. I_0 is the integrated ECL peak area of Ir complex at a concentration of 0.12 mM in the absence of the Ru complex, whereas I is the integrated ECL peak area of the Ir complex at a concentration of 0.12 mM in the presence of different concentrations of the Ru complex. The slope of the first three points of graph (a) is 957. Although this is lower than when including all points on this graph, is still much greater than the slopes calculated for the plots in graph (b).

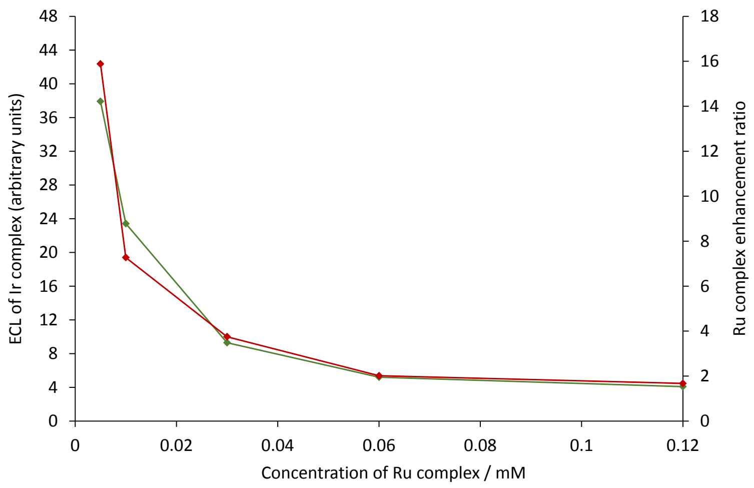

Figure S15. Green plot and left axis: the ECL intensity from 0.12 mM [Ir(df-ppy)$_2$(bpy)](PF$_6$) in the presence of various concentrations of [Ru(bpy)$_3$](PF$_6$)$_2$ (0.005 mM to 0.12 mM). Red plot and right axis: the ratio of the ECL from [Ru(bpy)$_3$](PF$_6$)$_2$ in the presence and absence of 0.12 mM [Ir(df-ppy)$_2$(bpy)](PF$_6$). Conditions as described in Fig. S13.

Synthesis and characterisation of metal complexes

NMR data was collected using a Varian FT-NMR 400 spectrometer or Varian FT-NMR 500 spectrometer (Varian, CA, USA). ^{1}H NMR spectra recorded at 400 or 500 MHz and $^{13}C\{^{1}H\}$ NMR spectra recorded at 101 MHz and 126 MHz respectively. Chemical shifts were referenced to residual solvent signals and quoted in ppm relative to tetramethylsilane (TMS). ^{19}F NMR spectra were collected at 470 MHz and chemical shifts were quoted relative to an internal standard of hexafluorobenzene (δ -164.9 ppm). ESI-MS spectra were recorded on an Agilent 6510 ESI-TOF LC/MS mass spectrometer (Agilent, CA, USA). Microwave reactions were carried out using a Biotage Initiator microwave reactor (Biotage, Uppsala, Sweden).

[Ir(df-ppy)$_2$(bpy)](PF$_6$)

Prepared by previously reported procedure[10] with some modifications. A mixture of [Ir[df-ppy]$_2$Cl]$_2$ (0.30 g, 0.25 mmol) and 2,2′-bipyridine (0.076 g, 0.49 mmol) in CH_2Cl_2/MeOH (30 mL, 2:1, v/v) was shielded from light and heated at reflux for 5 h. The mixture was cooled to ambient temperature and potassium hexafluorophosphate (0.10 g, 0.54 mmol) was added, then the mixture was shielded from light and stirred at ambient temperature for 2 days. The mixture was filtered to remove a colourless precipitate before removing the solvent by evaporation under reduced pressure. The crude product was loaded onto a silica gel column and eluted with CH_2Cl_2/acetone (15:1 v/v). The relevant fractions were collated and the solvent removed by evaporation under reduced pressure to afford a bright yellow solid (0.30 g, 0.34 mmol, 71%). ^{1}H-NMR (500 MHz; DMSO-d$_6$): δ 5.62 (dd, 2H, J = 8.4, 2.3 Hz), 6.97 (m, 2H), 7.24 (m, 2H), 7.71 (m, 4H), 7.93 (m, 2H), 8.04 (m, 2H), 8.31 (m, 4H), 8.90 (d, 2H, J = 8.2 Hz). ^{19}F NMR (470 MHz; DMSO-d$_6$): δ -106.52, -104.37, -67.97. ESI-MS (+ve ion) *m/z* $[M]^{+}$ 729.126 (experimental), 729.13 (calculated for $[C_{32}H_{20}F_4IrN_4]^{+}$).

[Ru(dtb-bpy)$_3$]Cl$_2$

A microwave vial (10-20 mL) charged with $[Ru(cod)Cl_2]_n$ (0.21 g, 0.75 mmol), 4,4′-di-*tert*-butyl-2,2′-bipyridine (0.61 g, 2.3 mmol) and dimethylformamide (10 mL) was sealed then heated to 150˚C (30 min) then at 160 ˚C (30 min). The reaction was cooled to ambient temperature and an orange precipitate was observed upon addition of diethyl ether. The precipitate was isolated by filtration, washed with diethyl ether then air dried to afford an orange powder (0.32 g, 0.33 mmol, 44%). ^{1}H NMR (400 MHz; CD_3CN): δ 8.55 (s, 6H, pyr*H*), 7.55 (m, 6H, pyr*H*), 7.39 (m, 6H, pyr*H*), 1.41 (s, 54H, *t*Bu*H*). ^{13}C NMR (101 MHz; $CDCl_3$): δ 163.4, 157.9, 151.7, 125.4, 122.8, 36.3, 30.5. ESI-MS (+ve ion) *m/z* $[M]^{2+}$ 453.308 (experimental), 453.24 (calculated for $[C_{54}H_{72}N_6Ru]^{2+}$).

TBA[Ir(ppy)$_2$(CN)$_2$]

TBA[Ir(ppy)$_2$(CN)$_2$] was prepared by modification to a previously reported procedure.[11] To a mixture of the dimeric iridium(III) complex [Ir(ppy)$_2$(Cl)]$_2$ (0.24 g, 0.22 mmol) in dichloromethane (30 mL), was added tetrabutylammonium cyanide (0.62 g, 2.3 mmol). The reaction mixture was heated at reflux for 19 h then the volume of the solvent was reduced under a stream of N_2. A yellow precipitate was observed after addition of petroleum spirits (boiling range: 40-60˚C, 60 mL). The precipitate was collected by filtration, washed with petroleum spirits and air-dried to afford a yellow powder (0.27 g, 0.34 mmol, 78%). ^{1}H NMR (400 MHz; DMSO-d$_6$): δ 9.54 (m, 2H, pyr*H*), 8.07 (d, $^{3}J_{HH}$ = 8.4 Hz, 2H, Ar*H*), 7.89 (m, 2H, pyr*H*), 7.66 (d, $^{3}J_{HH}$ = 7.6 Hz, 2H, pyr*H*), 7.30 (m, 2H, Ar*H*), 6.73 (m, 2H, pyr*H*), 6.61 (m, 2H, Ar*H*), 6.09 (m, 2H, Ar*H*), 3.16 (m, 8H, CH_2), 1.56 (m, 8H, CH_2), 1.30 (m, 8H, CH_2), 0.93 (t, $^{3}J_{HH}$ = 7.3 Hz, 12H, CH_3). $^{13}C\{^{1}H\}$ NMR (101 MHz; DMSO-d$_6$): δ 167.9, 164.0, 153.3, 144.4, 136.1, 130.78, 130.73, 128.2, 123.6, 122.5, 119.9, 118.9, 57.5, 23.1, 19.2, 13.5. ESI-MS (-ve ion) *m/z* $[M]^{-}$ 553.157 (experimental), 553.10 (calculated for $[C_{24}H_{16}IrN_4]^{-}$).

[Ru(dtb-ppy)$_3$][Ir(ppy)$_2$(CN)$_2$]$_2$

The [Ru(dtb-ppy)$_3$][Ir(ppy)$_2$(CN)$_2$]$_2$ salt was prepared by modification to a previously reported procedure.[12] A mixture of DCM (40 mL), [Ru(dtb-bpy)$_3$]Cl$_2$ (0.099 g, 0.10 mmol) and TBA[Ir(ppy)$_2$(CN)$_2$] (0.16 g, 0.20 mmol) was washed with water (7 × 20 mL) followed with brine (40 mL). The organic phase was dried ($MgSO_4$) and the solvent was removed. The product was recrystalised from a minimum of dichloromethane layered with diethyl ether to afford a red crystalline solid (0.065 g, 32 %). ^{1}H NMR (500 MHz; CD_3CN): δ 9.66 (m, 4H, pyr*H*), 8.51 (m, 6H, pyr*H*), 7.97 (m, 4H, Ar*H*), 7.85 (m, 4H, pyr*H*), 7.64 (m, 4H, pyr*H*), 7.58 (m, 6H, pyr*H*), 7.40 (m, 6H, pyr*H*), 7.21 (m, 4H, Ar*H*), 6.80 (m, 4H, pyr*H*), 6.70 (m, 4H, Ar*H*), 6.22 (m, 4H, Ar*H*), 1.42 (s, 54H, C*H*$_3$). ^{13}C{^{1}H} NMR (126 MHz; CD_3CN): δ 169.3, 165.1, 163.3, 157.9, 154.7, 151.7, 145.7, 137.0, 132.19, 132.09, 129.6, 125.5, 124.6, 123.6, 122.5, 121.3, 119.9, 36.3, 30.5. ESI-MS (+ve ion) *m/z* $[M]^{2+}$ 453.248 (experimental), 453.24 (calculated for $[C_{54}H_{72}N_6Ru]^{2+}$). ESI-MS (-ve ion) *m/z* $[M]^-$ 553.114 (experimental), 553.10 (calculated for $[C_{24}H_{16}IrN_4]^-$).

References

1. H. C. Moon, T. P. Lodge and C. D. Frisbie, *J. Am. Chem. Soc.*, 2014, **136**, 3705-3712.
2. A. Juris, V. Balzani, P. Belser and A. von Zelewsky, *Helv. Chim. Acta*, 1981, **64**, 2175-2182.
3. V. V. Pavlishchuk and A. W. Addison, *Inorg. Chim. Acta*, 2000, **298**, 97-102.
4. D. R. Whang, K. Sakai and S. Y. Park, *Angew. Chem., Int. Ed.*, 2013, **52**, 11612-11615.
5. A. Singh, K. Teegardin, M. Kelly, K. S. Prasad, S. Krishnan and J. D. Weaver, *J. Organomet. Chem.*, 2015, **776**, 51-59.
6. K. N. Swanick, M. Sandroni, Z. Ding and E. Zysman-Colman, *Chem. Eur. J.*, 2015, **21**, 7435-7440.
7. J. Li, P. I. Djurovich, B. D. Alleyne, M. Yousufuddin, N. N. Ho, J. C. Thomas, J. C. Peters, R. Bau and M. E. Thompson, *Inorg. Chem.*, 2005, **44**, 1713-1727.
8. K.-C. Chan, W.-K. Chu, S.-M. Yiu and C.-C. Ko, *Dalton Trans.*, 2015, **44**, 15135-15144.
9. D. Di Censo, S. Fantacci, F. De Angelis, C. Klein, N. Evans, K. Kalyanasundaram, H. J. Bolink, M. Grätzel and M. K. Nazeeruddin, *Inorg. Chem.*, 2008, **47**, 980-989.
10. M. Yu, Q. Zhao, L. Shi, F. Li, Z. Zhou, H. Yang, T. Yi and C. Huang, *Chem. Commun.*, 2008, 2115-2117.
11. M. K. Nazeeruddin, R. Humphry-Baker, D. Berner, S. Rivier, L. Zuppiroli and M. Grätzel, *J. Am. Chem. Soc.*, 2003, **125**, 8790-8797.
12. M. Sandroni and E. Zysman-Colman, *Dalton Trans.*, 2014, **43**, 3676-3680.
